# Hyaluronan Regulates Vascular Smooth Muscle Cell Osteogenic Differentiation and Vascular Calcification

**DOI:** 10.3390/biom16050729

**Published:** 2026-05-15

**Authors:** Shrea Roy, Jamie Kane, Irina Grigorieva, Dylan Roche-Dugmore, Sacha Moore, Robert Steadman, Anne-Catherine Raby, Lily Jakulj, Leon Schurgers, Esther Lutgens, Etto C. Eringa, Marc Vervloet, Donald Fraser, Soma Meran

**Affiliations:** 1 Wales Kidney Research Unit, Division of Infection and Immunity, School of Medicine, Cardiff University, Cardiff CF14 4XN, UK; 2 Vascular and Interventional Radiology Translational Laboratory, Department of Radiology, Mayo Clinic, Rochester, MN 55905, USA; 3 Department of Nephrology, Amsterdam University Medical Center, 1081 HV Amsterdam, The Netherlands; 4 Department of Biochemistry, Cardiovascular Research Institute Maastricht (CARIM), Maastricht University, Unversiteitssingel 50, 6229 ER Maastricht, The Netherlands; 5 Department of Cardiovascular Medicine and Immunology, Mayo Clinic, Rochester, MN 55905, USA; 6 Amsterdam Cardiovascular Sciences, Amsterdam University Medical Center, 1081 HV Amsterdam, The Netherlands; 7 Department of Nephrology, Radboud University Medical Center, 6525 GA Nijmegen, The Netherlands

**Keywords:** Hyaluronan, glycosaminoglycan, vascular calcification, vascular smooth muscle cells

## Abstract

Vascular calcification is a strong predictor of cardiovascular mortality and lacks effective treatment. The transformation of vascular smooth muscle cells (VSMCs) into osteoblast-like phenotypes is a key driver of calcification. This study identifies a regulatory role for Hyaluronan (HA) in VSMC osteogenic differentiation and arterial calcification. Human aortic VSMCs stimulated with high phosphate and/or pro-inflammatory cytokines (IL6 and TGF-β1) exhibited increased RUNX2, alkaline phosphatase and osteopontin expression, along with reduced contractility and elevated calcium deposition. This corresponded with reduced HA deposition and downregulation of HA synthase enzymes (HAS1, HAS2), Hyaluronidase enzymes (Hyal1), and HA binding proteins (CD44, TSG-6), whilst HAS3 and versican were upregulated. Comparable alterations in HA and protein expression were observed in an in vivo model of arterial calcification using vitamin K-deficient warfarin-fed mice. Pharmacological inhibition of HA synthesis, enzyme-mediated HA degradation and siRNA/plasmid modulation of HAS isoenzymes demonstrated a possible functional link between HA regulation and VSMC osteogenic differentiation. This study establishes HA and its associated binding proteins as key regulators of arterial calcification, highlighting a novel pathway for potential therapeutic intervention.

## 1. Introduction

Vascular calcification (VC) is highly prevalent in people with chronic kidney disease (CKD), diabetes, and the elderly, and it is associated with significant morbidity and mortality [1]. Established treatments for atherosclerotic cardiovascular disease such as antiplatelets, lipid-lowering therapy and percutaneous angioplasty are less effective in VC [2,3,4]. These suboptimal responses are underpinned by a unique vascular pathology, whereby VC in the arterial media is associated with vascular smooth muscle cell (VSMC) differentiation to an osteogenic phenotype [5,6]. VC has no therapy, reflecting an incomplete understanding of its pathophysiology. Thus, improved insights into mechanisms that drive VC are required before treatment strategies can be developed.

Hyaluronan (HA) is a non-sulphated matrix glycosaminoglycan that is ubiquitously expressed in somatic tissues in health and is altered during many pathologies. It is synthesised in humans by the HA synthase (HAS) enzymes 1, 2, and 3 and broken down by the hyaluronidase (Hyal) enzymes [7,8]. HA binding proteins (HABPs) refer to the large group of proteins that can bind HA, and specific HA-HABP interactions can affect cell state and function in divergent ways. In biological tissues the macromolecular structure of HA is dependent on its molecular weight and its interaction with receptors and proteins [7]. Thus, alterations in HA in association with various HABPs have been implicated in wide-ranging physiological and pathological processes including embryogenesis, development and ageing, tumorigenesis, inflammation, and fibrosis [9,10,11,12,13,14,15]. In the vasculature, HA has been shown to stimulate migration of endothelial cells and organise the capillary basal lamina [16]. *Has2* knockout mice demonstrate aberrant cardiac development and embryonic lethality, whilst *Has3* knockout mice are protected from atherosclerosis [10,17]. HA has also been implicated in the progression of atherosclerotic plaques and enhanced *Has2* expression promotes atherosclerosis indicating distinct roles for the HAS isoenzymes in vascular pathobiology [18,19]. Whilst there have been numerous studies examining the role of HA in intimal atherosclerotic disease [16,17,20,21,22], there has been limited research into its role in medial vascular calcification that is prominent in CKD, diabetes and the elderly.

The arterial tunica media consists of VSMCs in a framework of extracellular matrix. Calcification at this site is associated with the trans-differentiation of VSMCs to resemble bone-formative osteoblasts [5,23,24]. During this process VSMCs downregulate smooth muscle-specific genes such as alpha smooth muscle actin (α-SMA). In addition, they upregulate markers of osteogenesis such as runt-related transcription factor-2 (*RUNX2*), osteopontin, alkaline phosphatase, and secrete calcium-/phosphate-rich vesicles that contribute towards vessel mineralisation [5,24,25]. However, a better understanding of the mediators of VSMC phenotypic regulation is needed. HA has been identified as a critical regulator of cell phenotype and function, and our previous studies have identified a central role for HA in regulating Transforming Growth Factor-β1 (TGF-β1)-driven fibroblast differentiation and epithelial-to-mesenchymal transition [13,14,15,26,27,28,29]. Others have shown that HA is a key regulator of stem cell differentiation and cancer associated cell phenotypes [30,31,32,33,34,35]. However, the role of HA in regulating VSMC phenotype in the context of vascular calcification has not been explored. In this study we demonstrate that vascular calcification can be predisposed to and/or modulated via HA-dependent mechanisms and thus identify novel regulators of vascular calcification.

## 2. Materials and Methods

***Materials***—all reagents were purchased from Sigma-Aldrich (Poole, UK), Thermo Fisher Scientific (Waltham, MA, USA), BD Biosciences (San Jose, CA, USA) or GIBCO/Life technologies (Paisley, UK) unless otherwise stated.

***Cell culture****—*Primary human VSMCs were commercially purchased (Thermo Fisher, Gibco, UK) and grown in 35 mm dishes coated with bovine Fibronectin (PromoCell, Heidelberg, Germany) and incubated with smooth muscle growth medium: 5% foetal calf serum, 0.05 ng/mL recombinant human Epidermal Growth Factor, 0.5 ng/mL recombinant human Basic Fibroblast Growth Factor, 2 ng/mL recombinant human insulin, 100 units/mL penicillin, and 100 µg/mL Streptomycin. Cells were maintained at 37 °C in a humidified incubator in an atmosphere of 5% CO_2_, and fresh growth medium was added every 3–4 days.

***VSMC calcification****—*VSMC calcification was induced by culturing cells in growth medium supplemented with osteogenic medium (OM) as follows: 0.2 mM Ascorbic Acid 2-Phosphate, 10 mM Glycerol 2-Phosphate, 50 nM Dexamethasone and 10 mM sodium orthophosphate. This calcification medium was selected following a process of optimisation whereby this medium, a commercially available osteogenic medium (Thermo Fisher, UK), and an osteogenic medium containing 0.2 mM Ascorbic Acid 2-Phosphate, 10 mM Glycerol 2-Phosphate and 50 nM Dexamethasone only were evaluated for propensity to induce calcification at 21 days. Control cells were incubated in fresh smooth muscle medium alone. Fresh control or OM was replaced every 3 days.

***Cell Viability Assay***—Cells were counted using a LUNA-FLTM Automated Fluorescence Cell Counter (Logos Biosystem, Anyang, Republica of Korea). For each cell count, 10 μL of the cell sample was mixed with 10 μL of 0.4% Tryptan Blue Stain gently in a microcentrifuge tube by pipetting up and down. A total of 10–12 μL of the mixed sample was loaded into the inlet of one chamber of the counting slide LUNATM Reusable Slide (Cat # L12008 Logos Biosystem). The loaded slide was inserted into the slide port and “Bright Field Cell Counting” button was performed. After adjusting the focus of the cells displayed on the screen, “count” was selected and these generated data (Total, Live, and Dead cell concentrations, Viability, Average Cell Size, Actual number of Total, Live and Dead cells) were displayed.

***Cell stimulations and treatments*—**In selected experiments, VSMCs were stimulated with 10 ng/mL human recombinant TGF-β1 or 10 ng/mL IL-6 (R&D Systems, Abingdon, UK). In other experiments 0.5 mM 4-Methylumbelliferone (4MU) was used to inhibit HA synthesis, and 1 IU streptomyces hyaluronidase (Strep-Hyal) was used for enzymatic degradation of pericellular HA. 4MU is a coumarin derivative that chemically inhibits HA synthesis by depleting cellular uridine diphosphate glucuronic acid (UDP-GlcUA). Strep-Hyal cleaves HA at the β-D-GalNAc-(1-4)-β-D-GlcA bond yielding 3-(4-deoxy-β-D-gluc-4-enuronosyl)-N-acetyl-D-glucosamine tetra- and hexasaccharides.

***HAS1 and HAS2 plasmid over-expression****—*HAS1 and HAS2 open reading frame (ORF) was inserted into the plasmid pCR3.1, using standard ligation reactions with T4 DNA ligase (New England Biolabs, Ipswich, MA, USA). Amplification of the cloned vector was achieved via bacterial transformation into one-shot competent Escherichia coli and grown on ampicillin-containing agar. Single colonies were extracted, cloned and DNA-purified. Negative RT experiments were performed to ensure pCR3.1-HAS1 or HAS2 vectors were not conveying false positive over-expression. All samples were RQ1 DNAse-treated prior to RT to prevent amplification of ORF DNA. Transient transfection was performed using Lipofectamine LTX Transfection Kit according to manufacturer’s protocol (Life Technologies, Carlsbad, CA, USA). As a negative control, an empty pCR3.1 plasmid containing no ORF sequence was transfected.

***siRNA knockdown of Gene Expression****—*Transient transfection was carried out using specific siRNA nucleotides to HAS3 (ID 119475) according to the manufacturers protocol. As a control, cells were transfected with a negative control scrambled siRNA (I.D. AM4613) simultaneously (a scrambled sequence that bears no homology to the human genome).

***Animal experiments****—*VC in the arterial media is characteristic of CKD and is linked to vitamin K deficiency [36,37]. Warfarin is a vitamin K antagonist, and its use promotes medial VC [38]. Thus, an established murine model of warfarin-induced vitamin K deficiency causing medial VC was used [39,40]. DBA/2 mice were purchased from Charles River Laboratories Inc (Hertogenbosch, the Netherlands) and kept in a temperature-controlled environment (20 °C) with regular day/night cycles. Experiments were conducted under a licence approved by Maastricht University. A Warfarin diet was prepared as follows: warfarin (3 mg/g; supplemented with vitamin K1 1.5 mg/g) was mixed with normal chow diet (Arie Blok diets, Woerden, The Netherlands). Regular Western-type diet was bought directly from the supplier (0.25% cholesterol; Arie Blok diets, Woerden, the Netherlands). Mice were placed on warfarin diet for up to 4 weeks, and mice at 0 weeks were denoted as control. At sacrifice the aorta was flushed with 100 μM sodium nitroprusside, and the aortic arch and its main branches were excised and fixed in 1% paraformaldehyde and paraffin embedded.

***Alizarin Red assay****—*Alizarin Red staining was used as a determinant of calcium deposition. Cells were fixed with 4% paraformaldehyde for 15 min, then rinsed and 1 mL/well Alizarin Red solution added for 20 min. After washing, the plates were inspected with an inverted light microscope (Leica DMLA Light Microscope, Wetzlar, Germany) and representative images taken using an Olympus DP27 Microscope Digital Camera (Olympus, Tokyo, Japan). Dyed cells were analysed quantitatively for their calcium concentration by comparing OD_405_ values of acetic acid extracts with a panel of standard concentrations. A total of 400 µL of 10% acetic acid was added to wells for 30 min. A cell scraper was used to transfer the suspension to a microcentrifuge tube; the samples were vortexed and then heated to 85 °C for 10 min. Tubes were then transferred to ice for 5 min and centrifuged at 20,000× *g* for 15 min. Supernatant was then pH-neutralised. Alizarin Red standards were made by diluting 10× dilution buffer. Alizarin Red 2 mM working stock was used to set standards by using 2-fold serial dilutions. A total of 150 µL of standard samples were added to opaque 96-well plates and absorbance measured at OD_405_.

***Tissue calcium assays***—Alizarin Red S reacts with calcium cations to form a chelate and was used to directly stain for calcium in tissue sections. Mouse aorta sections were placed in a Techne Hybridisation Incubator (HB-1D) for 30 min at 60 °C. Deparaffinized sections were rehydrated in graded alcohols. Slides were rinsed and placed in a moistened slide chamber. A total of 2 g of Alizarin Red S was mixed with 100 mL of distilled water and the pH adjusted to 4.3 and then added to the tissue section for 5 min. Sections were dehydrated in Acetone-Xylene (1:1) solution and then rinsed with distilled water and graded alcohols before being mounted with Cytoseal. Tissue sections were inspected with a standard inverted light microscope. Representative images were taken using an Olympus DP27 Microscope Digital Camera. Von Kossa staining is also widely used to detect the presence of abnormal deposition of calcium phosphate in tissues; hence it was used as an additional stain to detect abnormal arterial mineralisation. Tissue sections were deparaffinized as above. Slides were incubated in 50 μL Silver Nitrate solution (5%) and exposed to UV light or 100-watt bulb for 1 h. Sections were washed in distilled water and 50 μL of 2% Sodium Thiosulphate for 5 min, counterstained with Nuclear Fast Red solution for 5 min and then mounted and viewed.

***Immunofluorescence and confocal microscopy****—*Cells were grown on 8-well Permanox chamber slides. They were fixed using 4% paraformaldehyde for 15 min and stained according to previous protocols [28]. Briefly, they were blocked with 5% bovine serum albumin for 20 min and then incubated with the primary antibody (Table 1) for 2 h. After further washing, slides were incubated with fluorescein isothiocyanate (FITC) conjugated and/or tetramethyl rhodamine isothiocyanate conjugated secondary antibodies for 1 h. Cell nuclei were stained with DAPI, and cells were mounted and analysed by Zeiss LSM880 (Carl Zeiss AG, Baden-Wurrtemberg, Germany) upright confocal microscopy with Airyscan. For tissue analysis, fixed mouse arteries were processed for embedding in paraffin and cut into 5 μm sections. Deparaffinized sections were rehydrated in graded alcohols and antigen retrieval performed in sodium citrate buffer in the autoclave at 120 °C for 20 min. Where biotinylated secondary antibodies were used, sections were incubated with avidin/Biotin block (Vector Laboratories) for 10 min. Sections were blocked in 10% goat serum (Vector Laboratories, Newark, CA, USA) for 20 min and incubated overnight at 4 °C with primary antibodies. Immobilised antibodies were detected by biotinylated secondary antibodies and AB reagents (Vector Laboratories) according to manufacturer’s instructions. For detection of HA, an HA binding protein (HABP, Merck (Darmstadt, Germany), cat.number 385911) with a biotin conjugate was used (1:200), followed by AB reagent or streptavidin conjugated with AlexaFluor 488/594 fluorophore. For immunofluorescence detection, AlexaFluor 488 goat anti-mouse IgG (H + L) and Alexa Fluor 594 goat anti-mouse IgG (H + L) secondary antibodies were used at 1:500 dilution (Table 2). DAPI (4′,6-diamidino-2-phenylindole) was used to visualise the nuclei. In negative controls, primary antibodies or HABP were omitted. HA localisation was assessed using streptomyces hyaluronidase-treated control samples in combination with confocal microscopy. Sections were imaged using Zeiss LSM880 Airyscan Confocal Laser Scanning Microscope.

***ImageJ analysis***—Regions of interest were selected using ImageJ software version 1.53 (NIH; Bethesda, MD, USA) for measurement of fluorescence intensity. Background fluorescence was subtracted by measurement of a nearby region without positive staining and then subtracting its Mean Grey Value from the ROI’s Mean Grey Value. For multiple ROI, this was repeated for each image to calculate the mean. The formulae used was corrected total cell fluorescence (CTCF) = Integrated Density − (Area of Selected Cell × Mean Fluorescence of Background readings).

***Reverse transcription and quantitative PCR (RT-qPCR)—***RT-qPCR was used to determine mRNA expression of osteopontin (*SPP1*), runt-related transcription factor 2 (*RUNX2*), alkaline phosphatase (*ALP*)**,** αSMA (*ACTA2*), HASs (*HAS1, HAS2, HAS3*), hyaluronidases (*HYAL1, HYAL2*), HA receptors (*CD44* and *RHAMM*), and HA binding proteins (*TSG6* and versican *(VCAN)*) according to established protocols [28]. Primers were either custom-designed or commercially available (Table 3). Total RNA was extracted using TRIzol. RT-qPCR was carried out using either TaqMan Fast Universal PCR master mix or Power SYBR Green PCR Master Mix (Thermo Fisher Scientific, Waltham, MA, USA). Samples were analysed on a ViiA 7 Real-Time qPCR System, and relative expression levels were normalised to a standard reference gene (18s rRNA) using the 2^−ΔΔCT^ method.

***Collagen gel-contractility assay—***Type I collagen was extracted from rat-tail tendon as previously described [41]. A total of 2.5 × 10^5^ VSMCs/mL was mixed into collagen lattice-forming solutions (2.5 mL 20% foetal calf serum–Dulbecco’s modified Eagle’s low-glucose medium, 500 μL 0.1 mol/L NaOH, and 2 mg/mL type I collagen). VSMC-populated collagen lattices were maintained at 37 °C, in 5% CO_2_ for 1 h, for collagen polymerization. They were detached and resuspended in serum-free medium containing appropriate treatments. The mean contraction values of the gel were obtained from by ImageJ software.

***HA ELISA****—*HA ELISA was commercially purchased (Corgenix, Broomfield, CO, USA) and used to assess concentrations of intracellular, cell-surface and extracellular HA in VSMC cultures according to established protocols [28]. Conditioned culture medium was removed and transferred into microcentrifuge tubes (soluble HA) on ice. Cells were then incubated with trypsin/EDTA for 5 min at RT, transferred into microcentrifuge tubes, and centrifuged at 4000× *g* for 5 min at 4 °C. Supernatant was transferred to microcentrifuge tubes; trypsin was deactivated by heating to 90 °C for 5 min (cell-surface HA) and then kept on ice. Cell pellets were resuspended in RIPA buffer and kept on ice for 10 min. The solution was centrifuged again, and supernatant transferred to fresh tubes (intracellular HA). Samples were then analysed by HA ELISA kits.

***Statistical analysis***—Statistical analysis was performed using Microsoft^®^ 365 Excel (Microsoft, Redmon, WA, USA) or GraphPad Prism 9.0 (Dotmatics, Boston, MA, USA). Graphical data were expressed as mean ± Standard Deviation (S.D). For statistical analysis across multiple experimental conditions, two-way Analysis of Variance (ANOVA) (parametric data) or Kruskal–Wallis with Dunn’s post hoc test (non-parametric data) were used to identify statistical differences across groups. *p* ≤ 0.05 was considered statistically significant (* *p* ≤ 0.05, ** *p* ≤ 0.01, *** *p* ≤ 0.001, **** *p* ≤ 0.0001).

## 3. Results

### 3.1. VSMCs Grown in Culture Supplemented with Increased Phosphate Upregulate Osteogenic Gene Expression and Deposit-Calcified Matrix, and This Leads to Impaired CELL Contractility

**Figure 1:**
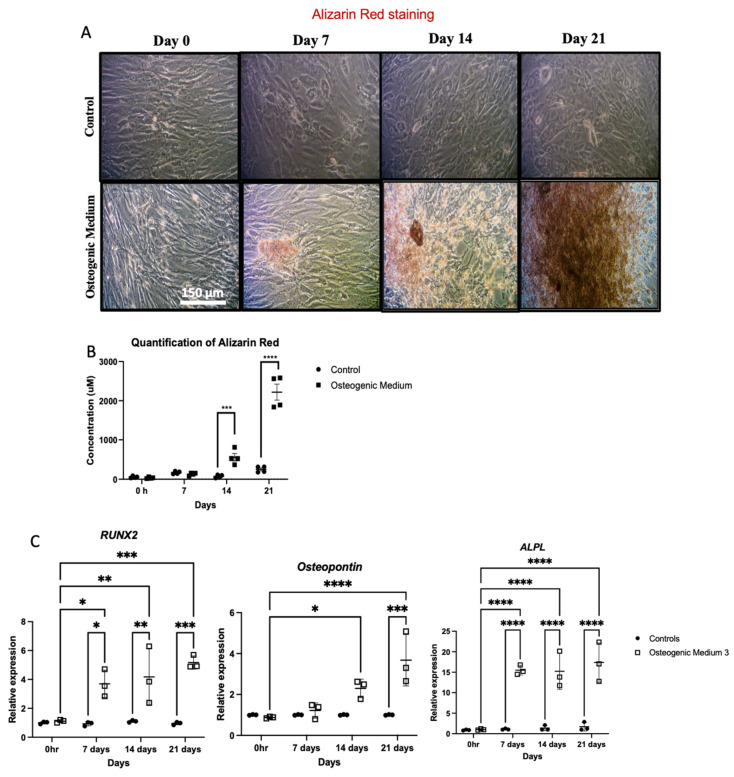
Human aortic vascular smooth muscle cells were grown in osteogenic medium, selected as described in the methods, for 0, 7, 14 and 21 days. Control cells were treated with VSMC growth medium for up to 21 days. (**A**) The media were replaced every 3 days. The cells were fixed and stained with Alizarin Red to pick up calcium deposition in the matrix, and the cells were analysed under a light microscope (original magnification ×100). (**B**) Alizarin Red was quantitatively measured using absorbance at OD405. (**C**) mRNA expression of *RUNX2*, osteopontin and alkaline phosphatase assessed by RT-qPCR. *β-actin* was used as a standard reference gene, and gene expression was assessed relative to control samples at 0 h. The comparative C_T_ method was used for relative quantification of gene expression, and the results are represented as mean ± S.D from three experimental repeats with statistical significance represented by: * *p* ≤ 0.05, ** *p* ≤ 0.01, *** *p* ≤ 0.001, **** *p* ≤ 0.0001.

**Figure 2:**
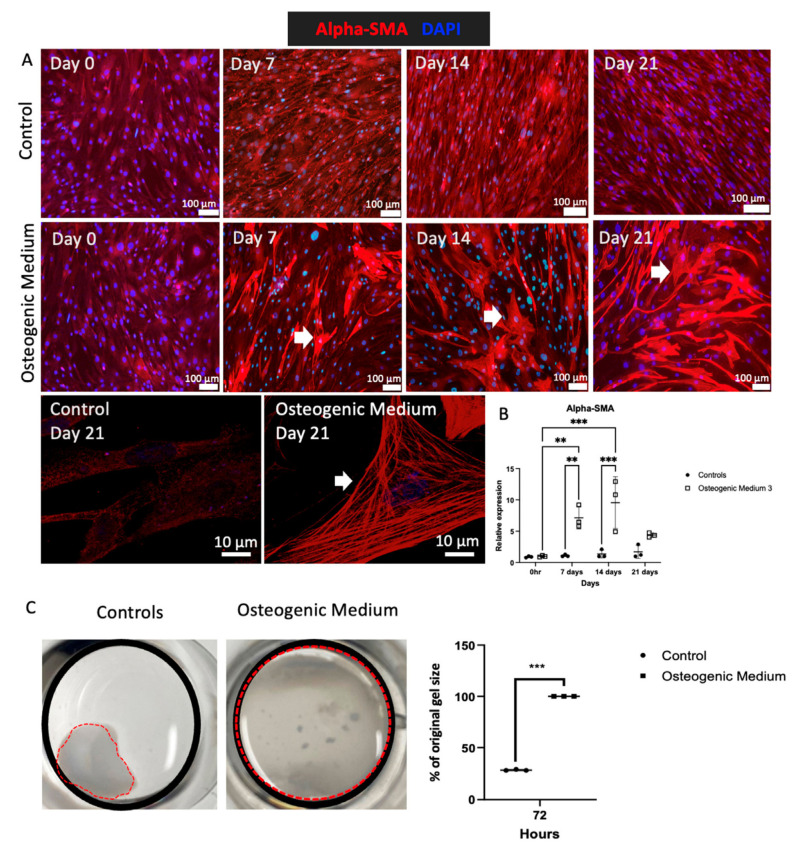
(**A**) Sub-confluent monolayers of human aortic vascular smooth muscle cells were grown with smooth muscle cell growth medium supplemented with osteogenic medium for 0, 7, 14 and 21 days. Control cells were grown in smooth muscle growth medium alone for up to 21 days. Cells were fixed using 4% paraformaldehyde and then stained for α-SMA (red). The cells were analysed by fluorescent microscopy (original magnification ×100 and ×400). White arrows denote the stress fibres. (**B**) mRNA expression of alpha-SMA was assessed by RT-qPCR. *β-actin* was used as a standard reference gene, and gene expression was assessed relative to control samples at 0 h. The comparative C_T_ method was used for relative quantification of gene expression and statistical analysis undertaken as previous. (**C**) VSMCs were seeded into pre-made collagen gels and grown to 50–60% confluence. Control cells were treated with VSMC growth medium. The collagen gels were photographed at 72 h and measured for analysis of rate of contraction. A representative photograph of one of three independent experiments is shown. The black circle shows the circumference of the well and the red dotted circle shows the circumference of the gels, denoting contraction. Statistical significance represented by: ** *p* ≤ 0.01, *** *p* ≤ 0.001.

Elevated serum phosphate is present in people with CKD and strongly correlates with medial VC in human studies [42]. Thus, experiments were performed on cultured VSMC grown in high phosphate media to investigate osteoblast-like transdifferentiation. VSMCs treated with high phosphate OM demonstrated increased calcium in the cell–matrix from 7 to 21 days (Figure 1A). Absorbance measurements confirmed this demonstrating significant increase in calcium staining following 14 and 21 days (Figure 1B). RT-qPCR assessed effects of OM on transcriptional regulation of osteogenic genes including *RUNX2*, osteopontin (*SPP1*) and alkaline phosphatase (*ALPL*) and demonstrated progressive increased expression of these genes (Figure 1C). VSMCs grown in control or lower sodium orthophosphate concentration (5 mM) demonstrated no discernible calcification (Supplementary Figure S1). α-smooth muscle actin (α-SMA, *ACTA2*) is a characteristic protein marker of VSMCs, which facilitates cell contractility. Cells stimulated with OM demonstrated altered morphology and increased α-SMA stress fibre formation (Figure 2A,B). VSMCs are typically highly contractile and regulate arterial compliance. However, when VSMCs underwent osteogenic transformation, their contractility was impaired despite their increased α-SMA expression (Figure 2C).

### 3.2. Cytokines Elevated in CKD Promote Osteogenic Gene Expression in VSMCs

**Figure 3:**
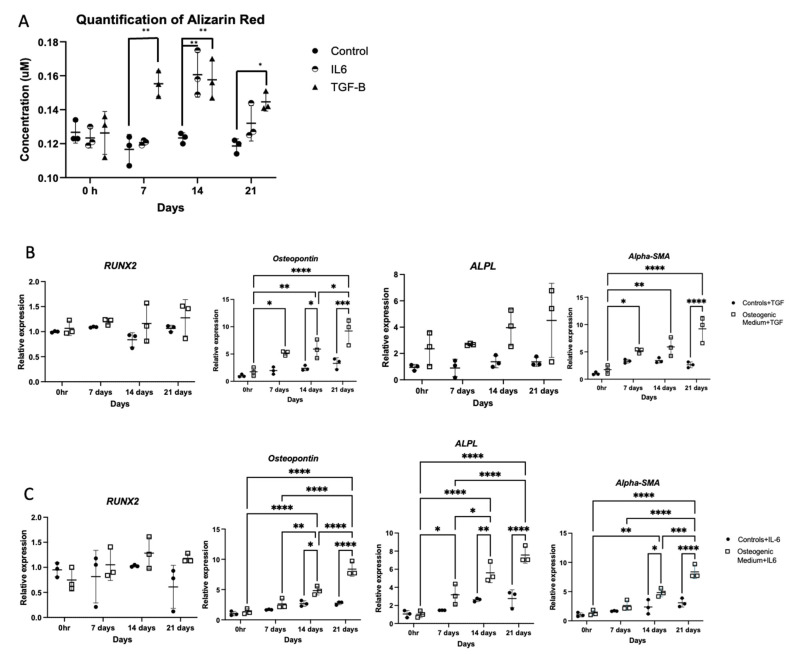
Sub-confluent monolayers of human aortic vascular smooth muscle cells were grown with smooth muscle cell growth medium supplemented with 10 ng/mL of IL-6 or 10 ng/mL of TGF-β1 for 0, 7, 14 and 21 days. Control cells were grown in smooth muscle growth medium alone for up to 21 days. (**A**) The cells were fixed and stained with Alizarin Red to pick up calcium deposition, and the cells with Alizarin Red were quantitatively measured using absorbance at OD405. (**B**,**C**) RT-qPCR was used to assess markers of osteogenic gene expression, and *β-actin* was used as a standard reference gene. The comparative C_T_ method was used for relative quantification of gene expression, and the results are represented as mean ± S.D from three experimental repeats with statistical significance represented by: * *p* ≤ 0.05, ** *p* ≤ 0.01, *** *p* ≤ 0.001, **** *p* ≤ 0.0001.

Previous studies established that Interleukin-6 (IL6) and Transforming Growth Factor-β1 (TGF-β1) are elevated in CKD [43]. Furthermore, increased levels of these cytokines correlate with increased VC in CKD [44]. Therefore, the direct effect of these cytokines in regulating VSMC-osteogenic transdifferentiation was explored. The results demonstrated increased calcium deposition at 7, 14 and 21 days, with IL6 and a trend towards increased calcium staining with TGF-β1 stimulation (Figure 3A). However, this was less pronounced than calcium deposition with OM. TGF-β1 promoted a significant increase in *SPP1* and *ACTA2* mRNA expression, whilst IL6 promoted increased *SPP1*, *ALPL* and *ACTA2* mRNA expression indicating that IL6 is a stronger promoter of osteogenesis compared to TGF-β1 (Figure 3B,C).

### 3.3. VSMC Osteogenic Transformation Is Associated with Marked Alterations in HA Matrix

**Figure 4:**
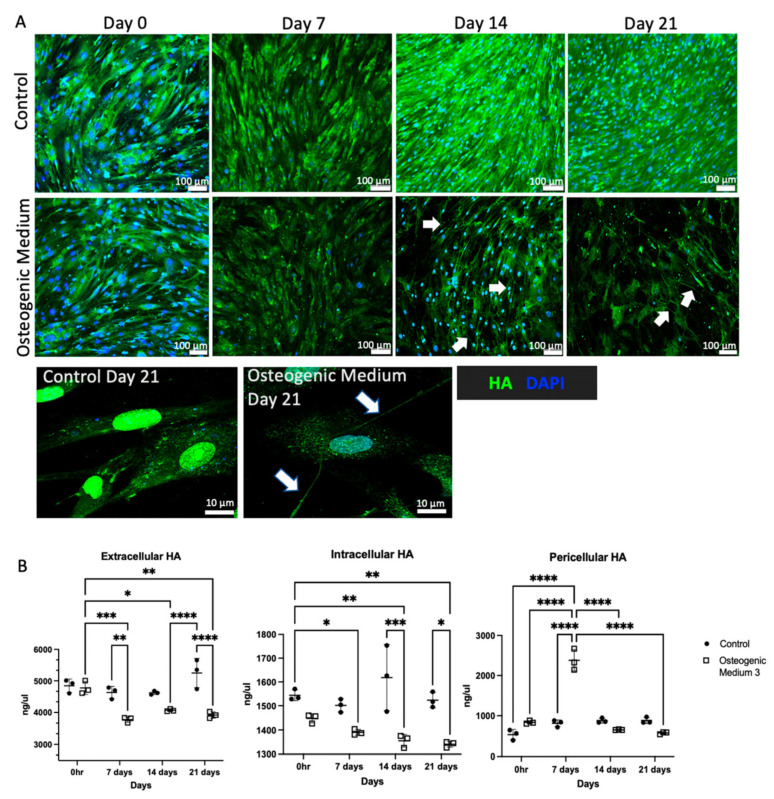
Human aortic vascular smooth muscle cells were grown in smooth muscle cell growth medium supplemented with osteogenic medium for 0, 7, 14 and 21 days. Control cells were grown in smooth muscle growth medium alone for up to 21 days. (**A**) Cells were fixed using 4% paraformaldehyde and stained for HA using biotinylated HABP (green) and analysed by fluorescent and confocal microscopy (original magnification ×100 and ×630). Individual filters can be seen in Supplementary Figure S8. (**B**) Quantification of extracellular, intracellular, and pericellular HA was undertaken by using a HA ELISA assay with statistical significance represented by: * *p* ≤ 0.05, ** *p* ≤ 0.01, *** *p* ≤ 0.001, **** *p* ≤ 0.0001.

HA matrix in normal and osteogenic VSMCs was initially assessed using immunofluorescence. In control VSMCs, HA was diffusely expressed in the cytoplasm and in/around the nucleus. Incubation with OM led to marked morphological change in VSMCs, from “spindle-shaped” cells to “poached-egg”-shaped cells. This was associated with reduced cytoplasmic HA staining and increased pericellular HA strands interconnecting cells (white arrows). The overall cellular intensity of HA staining was attenuated as VSMCs underwent osteogenic differentiation (Figure 4A). HA ELISA was performed to quantify levels of intracellular, pericellular and extracellular HA and confirmed that osteogenic transformation led to overall attenuation of extracellular and intracellular HA. However, there was a significant increase in pericellular HA 7 days following OM stimulation (Figure 4B).

**Figure 5:**
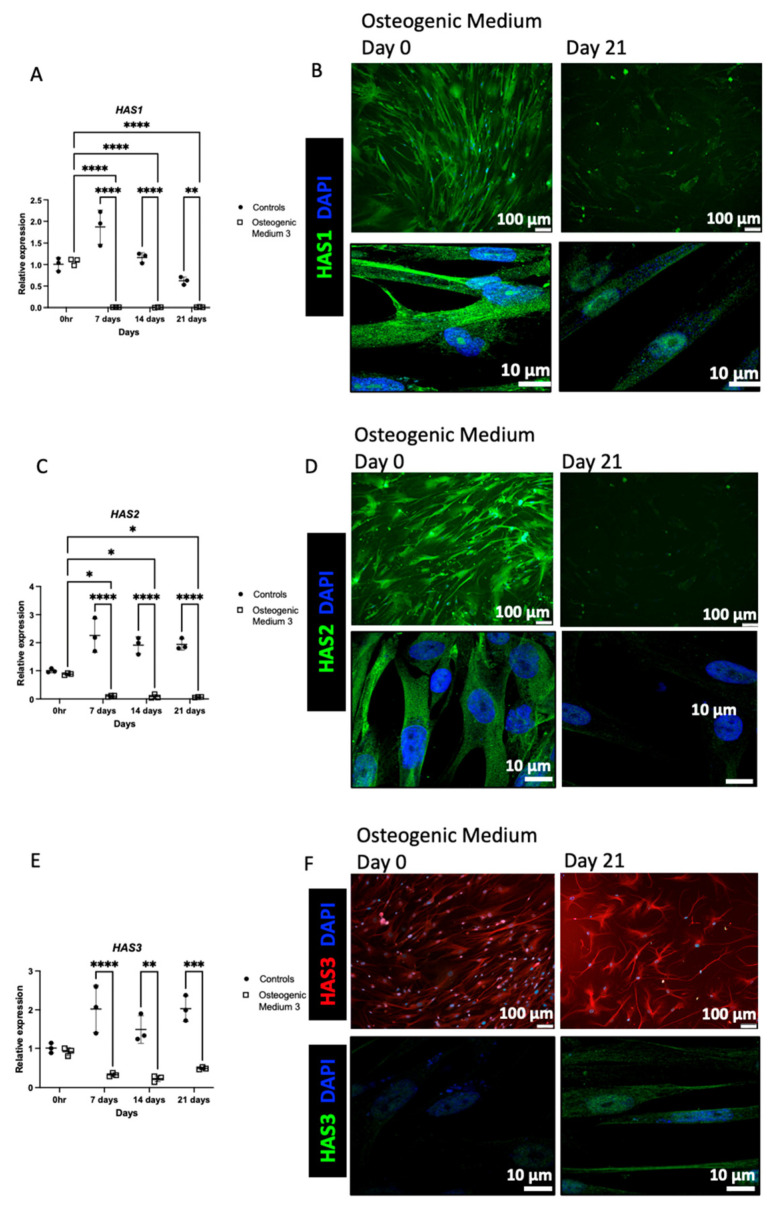
Human aortic vascular smooth muscle cells were grown in VSMC growth medium alone or VSMC growth medium supplemented with osteogenic medium for up to 21 days. In (**A**,**C**,**E**) RT-qPCR was used to measure mRNA expression of *HAS1*, *HAS2* and *HAS3* at 0, 7, 14 and 21 days. The graphs represent three separate experiments using data from confocal (lower) images. The results are represented as mean ± S.D from three experimental repeats with statistical significance represented by: * *p* ≤ 0.05, ** *p* ≤ 0.01, *** *p* ≤ 0.001, **** *p* ≤ 0.0001. In (**B**,**D**,**F**) cells were fixed using 4% paraformaldehyde and then stained for HAS1 (green), HAS2 (green), HAS3 (red) and DAPI (blue). The cells were analysed by confocal microscopy, magnification ×100 and ×630.

**Figure 6:**
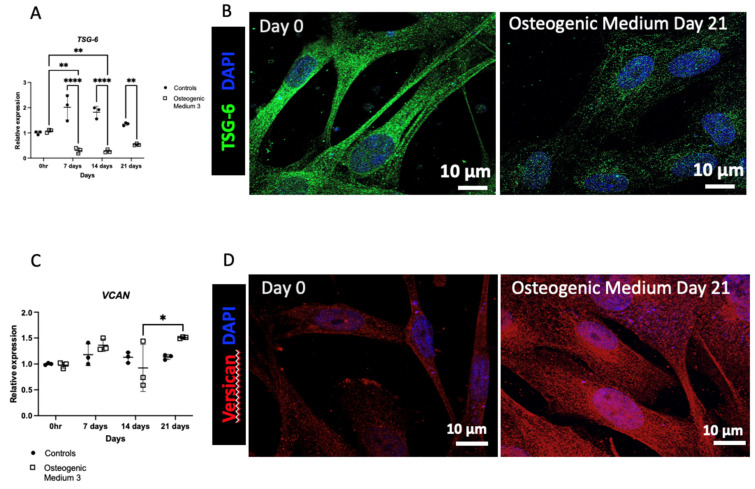
Human aortic vascular smooth muscle cells were grown in VSMC growth medium alone or VSMC growth medium supplemented with osteogenic medium for up to 21 days. In (**A**,**C**), RT-qPCR was used to measure mRNA expression of *TSG-6* and *VCAN* at 0, 7, 14 and 21 days. The graphs represent three separate experiments. The results are represented as mean ± S.D from three experimental repeats with statistical significance represented by: * *p* ≤ 0.05, ** *p* ≤ 0.01, **** *p* ≤ 0.0001. In (**B**,**D**) cells were fixed using 4% paraformaldehyde and then stained for TSG-6 (green), versican (red) and DAPI (blue). The cells were analysed by confocal microscopy, original magnification ×630.

HA is generated by three HA synthase (HAS) isoenzymes (HAS1, HAS2, and HAS3). In VSMCs, HAS1 and then HAS2 were the most abundant isoenzymes (C_T_ values 20 and 22 respectively), with lower expression of HAS3 (C_T_ value 28). Following osteogenic differentiation HAS1 and HAS2 mRNA and protein expression were markedly attenuated (Figure 5A–D). The corresponding C_T_ values are >33 for HAS1 and >29 for HAS2. In contrast, whilst HAS3 mRNA expression was also initially attenuated, there was a subsequent increase in HAS3 mRNA at days 14 and 21 (C_T_ values 27). Furthermore, HAS3 protein expression was increased following osteogenic differentiation (Figure 5E,F and Supplementary Figure S2). Similarly, stimulation with IL6 led to attenuated HAS1 and HAS2 expression and enhanced HAS3 expression (Supplementary Figure S3). Expression of the HA catalytic enzyme: HYAL1 was attenuated along with attenuation of the intracellular HA receptor, RHAMM (Receptor for HA-Mediated Motility). Expression of the other HA catalytic enzymes and receptors (HYAL2 and CD44) demonstrated no significant alterations (Supplementary Figure S4). HA can bind to a multitude of proteins termed HA-binding proteins (HABPs). In view of the observed alterations in pericellular HA, expression of the following two common cell-surface HABPs known to regulate HA macromolecular structure were investigated: versican and TSG-6 (*TNFAIP6*, Tumour Necrosis Factor Stimulated Gene-6). VSMCs that underwent osteogenic differentiation demonstrated attenuated *TNFAIP6* mRNA and protein expression, whilst exhibiting enhanced versican expression (Figure 6). Interestingly, stimulation of VSMCs with both IL6 and TGF-β1 also increased versican expression (Supplementary Figure S5).

### 3.4. There Is a Functional Link Between HA Modulation and Osteogenic Transformation in VSMCs

**Figure 7:**
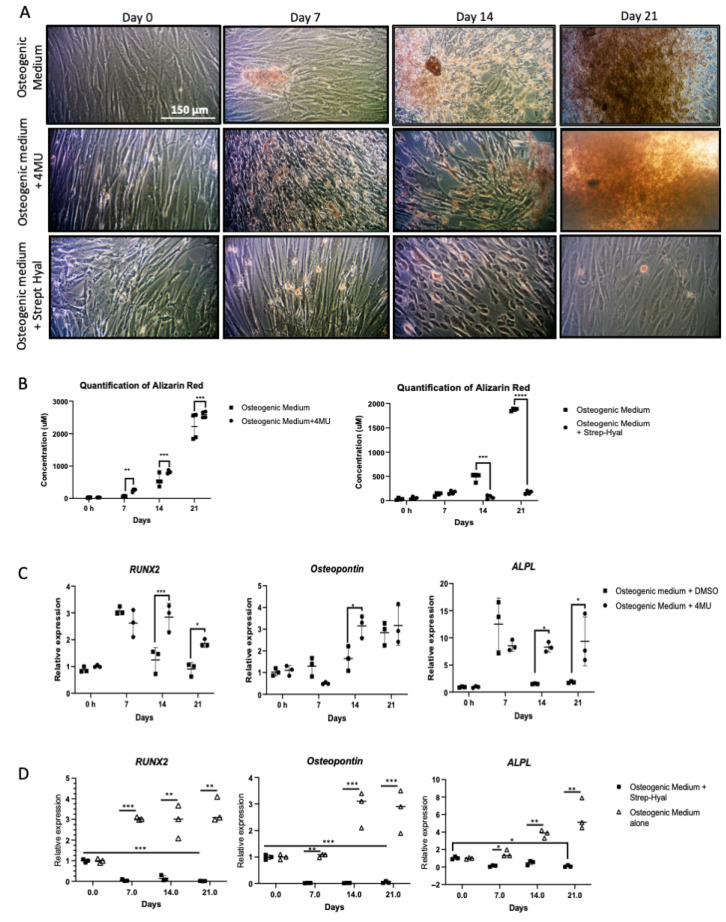
Human aortic vascular smooth muscle cells were grown in VSMC growth medium supplemented with osteogenic medium alone or osteogenic medium supplemented with 4MU or streptomyces hyaluronidase for 0, 7, 14 or 21 days. (**A**) The cells were fixed and stained with Alizarin Red and examined under a light microscope (original magnification ×100). (**B**) The cells were extracted for quantitative measurement of absorbance at OD405. (**C**,**D**) mRNA expression of the osteogenic markers *RUNX2*, osteopontin and alkaline phosphatase was assessed by RT-qPCR with the results represented as mean ± S.D from three experimental repeats with statistical significance represented by: * *p* ≤ 0.05, ** *p* ≤ 0.01, *** *p* ≤ 0.001, **** *p* ≤ 0.0001.

Depletion of the UDP-glucuronic acid pool by 4MU was used to inhibit HAS enzyme function and determine the effects of this on VSMC osteogenic phenotype. In parallel, the effect of targeted degradation of pericellular HA matrices was undertaken by incubation of VSMCs with Strep-Hyal. Both these methods are widely established in inhibiting HA synthesis and specific degradation of pericellular and extracellular HA matrix respectively [45,46,47]. The results demonstrated that whilst global inhibition of HA synthesis with 4MU led to enhanced calcification, degradation of HA pericellular matrices with Strep-Hyal led to markedly attenuated calcification in VSMC osteogenic cultures (Figure 7A,B). Effects of 4MU and Strep-Hyal on VSMC osteogenic gene expression showed similar findings with 4MU promoting enhanced *RUNX2*, *SPP1* and *ALPL* expression, whilst Strep-Hyal attenuated expression of these osteogenic markers (Figure 7C,D).

**Figure 8:**
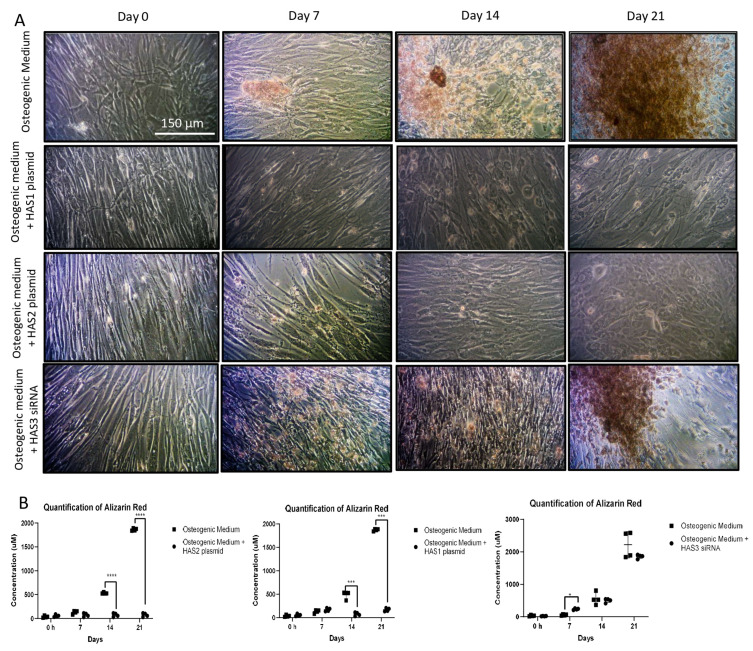
Human aortic vascular smooth muscle cells were transfected with either an empty vector (pCR 3.1) or a vector containing either *HAS1*, or *HAS2* plasmid DNA or *HAS3* siRNA for 72 h. The cell medium was then supplemented with osteogenic medium for 0, 7, 14 or 21 days. (**A**) The cells were fixed and stained with Alizarin Red and examined under a light microscope (original magnification ×100). (**B**) The cells were extracted for quantitative measurement of absorbance at OD405 with statistical significance represented by: * *p* ≤ 0.05, *** *p* ≤ 0.001, **** *p* ≤ 0.0001.

The distinct HAS isoenzymes are located on different chromosomes, are conserved in evolution, and have differing levels of activity [48]. Thus, distinct HAS isoenzymes may have different roles in VSMC differentiation. In our data HAS1 and HAS2 mRNA and protein expression were attenuated following VC, whilst HAS3 expression was increased. To explore this, isolated *HAS1* versus *HAS2* over-expression in VSMCs was undertaken and compared to the effects of *HAS3* knockdown. Enhanced *HAS1* and *HAS2* expression led to a significant reduction in calcification. By comparison, *HAS3* abrogation did not demonstrate a statistically significant change (Figure 8). Similarly, there was notable reduction in *RUNX2*, *SPP1* and *ALPL* mRNA following *HAS1* and *HAS2* over-expression. In contrast, *HAS3* knockdown led to an increase in *RUNX2*, *SPP1* and *ALPL* at day 7, but there was no discernible difference in osteogenic gene expression following 14 and 21 days (Supplementary Figure S6).

### 3.5. Arterial Changes in HA Matrix Are a Prominent Feature in the Development of VC In Vivo

**Figure 9:**
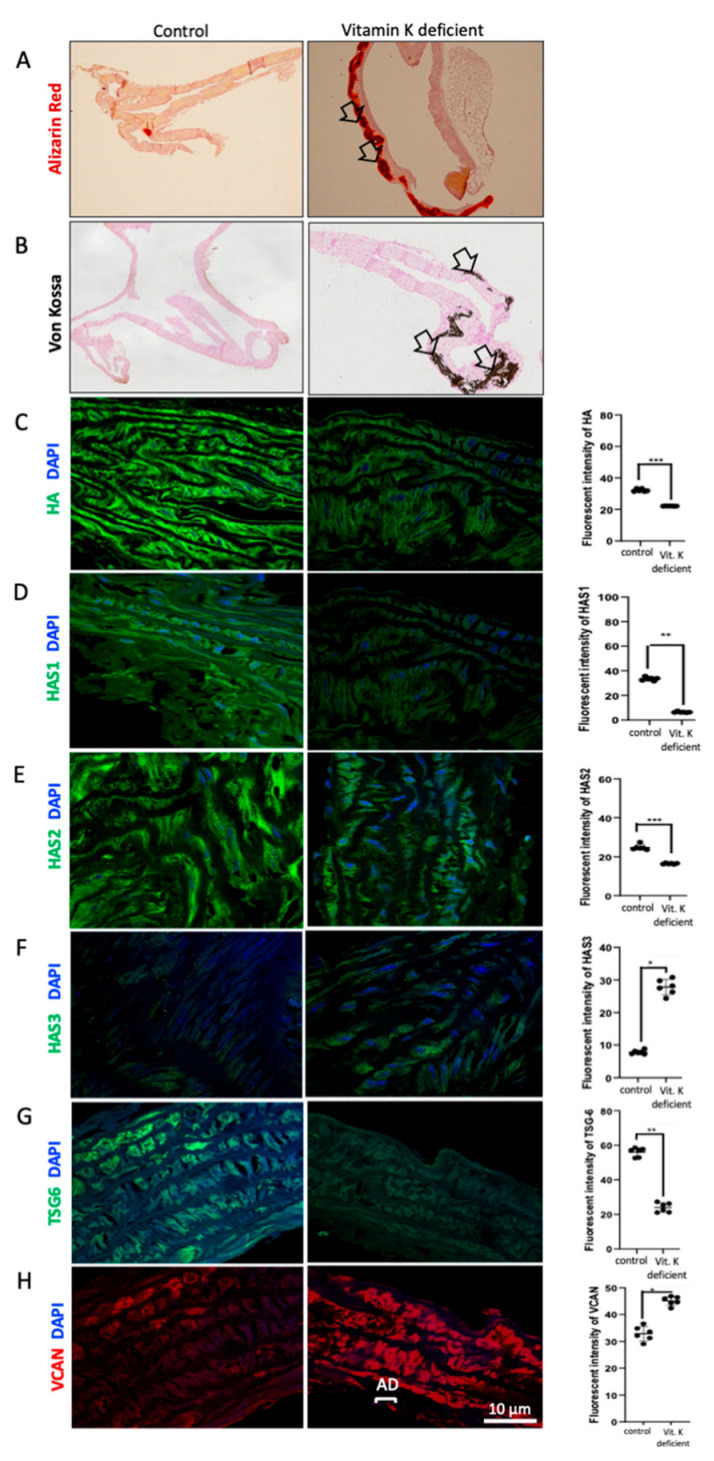
DBA/2 mice were fed with 3mg/g of warfarin mixed with normal chow diet for up to 4 weeks. The mice were then sacrificed and aorta harvested fixed in 1% paraformaldehyde for analysis. Sections were subsequently stained for (**A**) Alizarin Red, (**B**) Von Kossa, (**C**) HA, (**D**) HAS1, (**E**) HAS2, (**F**) HAS3, (**G**) TSG-6 or (**H**) versican. DAPI was used as the nuclear stain. Images were taken using Laser Point scanning confocal microscopy and the fluorescent intensity measured using ImageJ software. Original magnification ×400 and scale bars represent 10 μm. All the values are expressed as means ± SD from 6 aorta sections in each group with statistical significance represented by: * *p* ≤ 0.05, ** *p* ≤ 0.01, *** *p* ≤ 0.001.

Arterial mineralisation was assessed in the aorta of normal versus vitamin K-deficient mice using Alizarin Red and Von Kossa staining, both widely used to detect the presence of abnormal calcium deposits in tissues. Alizarin Red and Von Kossa staining both revealed that vitamin K-deficient animals displayed significant calcium mineralisation in the arterial media compared to control animals (Figure 9A,B). Immunofluorescent staining detected abundant HA staining in the arterial media in control mice. In vitamin K-deficient mice there was marked HA attenuation in the arterial media (Figure 9C). Both HAS1 and HAS2 staining was attenuated in the arterial media of vitamin K versus normal mice (Figure 9D,E). HAS3 staining was low compared to HAS1 and HAS2 staining in normal mice arteries; however, HAS3 was enhanced in vitamin K-deficient mice (Figure 9F). In keeping with the in vitro findings, expression of TSG-6 was attenuated and expression of versican enhanced in media in vivo (Figure 9G,H). Cell viability assays were undertaken with all experiments and demonstrated no significant alterations in cell numbers following osteogenic stimulation (Supplementary Figure S7).

## 4. Discussion

The pathogenesis of VC is increasingly recognised to be a complex cell-regulated process, beyond a simple precipitation of increased levels of calcium and phosphate. From the previous literature it is evident that VSMCs exhibit phenotypic plasticity and can display a spectrum of phenotypes [49]. In CKD, VSMCs are prone to osteogenic differentiation, and this strongly correlates with high circulating levels of serum phosphate and increased systemic inflammation, particularly elevated levels of IL6 and TGF-β1 [5,43,44]. However, the mediators that link high phosphate and systemic inflammation to changes in VSMC phenotype are poorly understood. In this study we have shown a link between inflammation and VC and alterations in HA matrix.

Previous studies have shown the importance of ECM in regulating cell phenotype and function [10,13,26,28,29]. In particular, HA matrix has been identified as a key regulator of cell phenotype in many biological contexts. Furthermore, studies have also shown that increased systemic inflammation and cytokine changes drive significant changes in the expression and organisation of HA matrix and its related binding proteins [13,50,51]. In this study we hypothesised that HA alterations may also be an important regulator of VSMC osteogenic transdifferentiation. The results in this paper confirmed that elevated phosphate and elevated cytokines (IL6 and TGF-β1) promoted considerable changes in HA metabolism in VSMCs and this was associated osteogenic transformation of these cells. Furthermore, we demonstrated from the in vitro studies that there was a possible causal link between changes in HA matrix and the ability of the cells to undergo osteoblast-like transdifferentiation.

As with much research related to ECM, some of the presented data related to causality portrays complexity and seemingly contradictory results. Whilst inhibition of HA synthesis promoted osteoblast-like transition of VSMCs and increased calcium mineralisation in the matrix, enzymatic digestion of pericellular HA matrices protected against VSMC-osteoblastic differentiation and calcification. This indicates that it may not be simply the presence versus absence of HA molecules alone that influences calcification. We know from the literature that alterations in HA can lead to biologically diverse outcomes depending on the manner of its synthesis, breakdown, molecular weight and macromolecular organisation, which is dependent on its interaction with a multitude of HA binding proteins [26,27,28]. In this study the results demonstrated that the predominance of specific iso-enzymes involved in HA synthesis played an important role in calcification outcomes. Whilst HAS1 and HAS2 expression favoured normal VSMC phenotype and reduced calcification, the role of the HAS3 iso-enzyme was less clear with some suggestion that it may favour calcification. It has been reported that HAS3 produces lower molecular weight HA, while HAS1 and HAS2 make higher molecular weight HA, and molecular weights of HA dictate interaction with different hyaluronidases [52,53]. The definitive role of all HAS iso-enzymes and their associated HA molecular weights in medial arterial calcification will require further in vivo investigation and modulation. A previous study on HAS2 transgenic mice demonstrated that HA accumulation increased vessel stiffness and promoted a synthetic VSMC phenotype and altered ECM composition, contributing to atherosclerotic priming by enhancing cholesterol retention and inflammation [54]. While the study suggested HA may prime the vasculature for calcification, calcification was not actually assessed. However, the combined findings from these studies indicate that HA regulation is paramount to normal vascular biology, atherosclerosis and calcification, highlighting the need for research in models that combine both VC and atherosclerosis.

HA is a simple linear non-sulphated GAG consisting of repeating disaccharide units of the two sugars N-acetyl glucosamine and D-glucuronic acid. However, it demonstrates diverse biological roles attributed to its propensity to bind to a multitude of HA binding proteins (HABPs), termed hyaladherins [7,8]. HA–hyaladherin interactions can influence the macromolecular organisation of cell-associated HA. We and others have shown that cells can form “HA coat” or “HA cable-like” structures influenced by the balance of tissue expression and affinity of various HA–hyaladherin interactions, and these macromolecular structures promote distinct HA-dependent biological functions [26,51,55,56]. Versican and TSG-6 are two widely expressed hyaladherins in vertebrates that have been well studied. TSG-6 has been identified as a critical regulator of HA coats, and versican as a key constituent of HA cables [26,55,56]. The in vivo and in vitro studies presented in this manuscript suggest that versican and TSG-6 may play key but opposing roles in the regulation of VSMC phenotype and medical VC, with increased TSG-6 favouring normal VSMC phenotype and increased versican favouring VSMC osteoblast-like transformation and vascular mineralisation. Furthermore, alongside increased versican expression, VSMCs that adopted an osteoblast-like phenotype appeared to form HA cable-like structures in the cell–matrix. These HA cables have previously been shown to promote inflammation through enhancing monocyte–cell interactions and sequestration of inflammatory cells in tissues [57]. This may point to a potential mechanism through which versican-dependent HA cables may promote inflammation-driven VC. Interestingly, a previous study has also shown a specific link between HAS3 and formation of HA cables [58]. In conjunction, these findings suggest that pericellular organisation of HA matrix, driven by its composition and binding to various HABPs, may be a key regulator of the calcification process and requires further exploration in interventional animal studies. Other work has shown that HA molecular weight is critical in regulating calcification during bone and cartilage formation, and this may play a similar role in vascular calcification [59]. HA molecular weight analysis in VC has not been undertaken in this study but represents an important avenue for future research.

The main challenge in conducting this study was identifying the most suitable model to study arterial calcification relevant to CKD. Individuals with CKD are specifically prone to medial vascular calcification and often exhibit subclinical vitamin K deficiency [37]. Furthermore, high vitamin K intake led to a reduction in coronary disease and aortic calcification in clinical studies [59]. In line with this, our animal studies have shown that vitamin K deficiency specifically leads to medial artery calcification. Thus, the model of warfarin-induced vitamin k deficiency was chosen to investigate the mechanisms involved in VSMC-driven medial calcification. However, it is acknowledged that this is a model that does not overtly exhibit systemic inflammation, which correlates strongly with medial VC, and this was largely inferred from the in vitro studies with IL6 and TGF-β1. Thus, further study will focus on adaptation of the in vivo model to better account for CKD-associated systemic inflammation. Although the precise mechanisms through which IL-6 and TGF-β1 promote vascular calcification were not directly examined in this study, these cytokines likely act through distinct but complementary pathways. IL-6 may promote inflammatory osteogenic signalling through STAT3-associated mechanisms, whereas TGF-β1 is linked to MAPK and SMAD-dependent matrix remodelling and VSMC phenotypic switching. Convergence of these pathways may contribute to medial vascular calcification in CKD, where chronic inflammation and fibrosis coexist.

## 5. Conclusions

In conclusion the findings presented in this study suggest that modulation of HA matrix may present a novel approach to regulation of VSMC phenotype and calcification in the arterial media. We propose that HA generated through the HAS1 and HAS2 isoenzymes and linked with TSG-6 may be protective of the development of VC. In contrast, HA generated through the HAS3 iso-enzymes and linked with versican and HA cable-like structures does not protect against VC. The study overall highlights the need for exploring the development of stromal-targeted therapies in this field.

## Figures and Tables

**Table 1:** List of primary antibodies with concentration.

Antibody	Host and Type	Dilution
Anti-α-SMA	Polyclonal–Mouse/IgG2a	1:25
Phalloidin—Conjugate Alexa Fluor 555 (Sigma)	-	1:25
Hyaluronan Binding Protein (HABP) (Merck Lifesciences)	-	1:100
Anti-HAS1 (Abcam, Cambridge, UK)	Monoclonal–Rabbit	1:100
Anti-HAS2 (Santa Cruz, Dallas, TX, USA)	Monoclonal–Rabbit	1:100
Anti-HAS3	Polyclonal–Rabbit/IgG	1:100
Anti-HYAL1	Polyclonal–Rabbit/IgG	1:100
Anti-HYAL2	Monoclonal–Goat	1:100
Anti-CD44	Monoclonal–Rabbit	1:100
Anti-RHAMM	Polyclonal–Rabbit/IgG	1:100
Anti-TSG-6	Polyclonal–Rabbit/IgG	1:100
Anti-VCN	Polyclonal–Mouse/IgG	1:100

**Table 2:** List of secondary antibodies with concentration.

Antibody	Host and Type	Dilution
Anti-Mouse-IgG2b (Alexa Fluor 488)	Polyclonal-Goat	1:500
Anti-Rat-IgG1 (Alexa Fluor 488)	Polyclonal-Goat	1:500
Anti-Rabbit-IgG (Alexa Fluor 488)	Polyclonal-Goat	1:500
Anti-Mouse-IgG2a (Alexa Fluor 594)	Polyclonal-Goat	1:500
Anti-Rat-IgG (Alexa Fluor 594)	Polyclonal-Goat	1:500
Anti-Rabbit-IgG (Alexa Fluor 594)	Polyclonal-Goat	1:500
Streptavidin 488	N/A	1:200
Streptavidin 594	N/A	1:200

**Table 3:** List of primers.

Gene Target	TaqMan^®^ Gene Expression Assay
*RUNX2*	Hs01047973_m1
*SPP1*	Hs00959010_m1
*ALPL*	Hs01029144_m1
*ACTA2*	Hs00426835_m1
*HAS1*	Hs00987418_m1
*HAS2*	Hs00193435_m1
*HAS3*	Hs00193436_m1
*HYAL1*	Hs00537920_g1
*HYAL2*	Hs01117343_g1
*CD44*	Hs00153304_m1
*RHAMM*	Hs00234864_m1
*TSG-6*	Hs00200180_m1
*VCAN*	Hs00171642_m1
*Β* *-Actin*	Hs01060665_g1

## Data Availability

The original contributions presented in this study are included in the article/Supplementary Materials. Further inquiries can be directed to the corresponding author.

## Supplementary Materials

The following supporting information can be downloaded at https://www.mdpi.com/article/10.3390/biom16050729/s1, Figure S1. Low phosphate versus high phosphate medium for inducing calcification in vitro. Figure S2. HAS3 protein expression is increased following osteogenic differentiation. Figure S3. The effect of IL-6 and TGF- β1 stimulation on HAS isoenzyme expression in vitro. Figure S4. No significant changes in HYAL2 and CD44 during osteogenic differentiation. Figure S5. Versican expression is increased by stimulation with both IL-6 and TGF-β1. Figure S6. Changes in osteogenic markers following HAS1 and HAS2 over-expression and HAS3 knockdown. Figure S7. Cell viability assays demonstrate no significant alterations in cell numbers following osteogenic stimulation. Figure S8. Supplement to figure (Figure 4)—DAPI and HA filters individually.

## Abbreviations

The following abbreviations are used in this manuscript:

α-SMA	Alpha Smooth Muscle Actin
ALPL	Alkaline Phosphatase
CD44	Cluster of Differentiation 44
CKD	Chronic Kidney Disease
HA	Hyaluronan
HABP	Hyaluronan Binding Proteins
HAS	Hyaluronan Synthase
HYAL	Hyaluronidase
IL-6	Interleukin 6
4MU	4-Methylumbelliferone
OM	Osteogenic Medium
ORF	Open Reading Frame
RHAMM	Receptor for HA-Mediated Motility
RUNX2	Runt-Related Transcription Factor 2
TGF-β1	Transforming Growth Factor-β1
TSG6	Tumour Necrosis Factor-Inducible Gene-6
VSMC	Vascular Smooth Muscle Cell

## References

[1] Go A.S., Chertow G.M., Fan D., McCulloch C.E., Hsu C.Y. (2004). Chronic kidney disease and the risks of death, cardiovascular events, and hospitalization. N. Engl. J. Med..

[2] Bansal N., Hsu C.Y., Chandra M., Iribarren C., Fortmann S.P., Hlatky M.A., Go A.S. (2011). Potential role of differential medication use in explaining excess risk of cardiovascular events and death associated with chronic kidney disease: A cohort study. BMC Nephrol..

[3] Shlipak M.G., Fried L.F., Cushman M., Manolio T.A., Peterson D., Stehman-Breen C., Bleyer A., Newman A., Siscovick D., Psaty B. (2005). Cardiovascular mortality risk in chronic kidney disease: Comparison of traditional and novel risk factors. JAMA.

[4] Charytan D.M., Kuntz R.E., Chhabra A., Cutlip D.E. (2006). Relationship of chronic kidney disease to cardiovascular death and myocardial infarction following coronary stenting. J. Nephrol..

[5] Paloian N.J., Giachelli C.M. (2014). A current understanding of vascular calcification in CKD. Am. J. Physiol. Ren. Physiol..

[6] Shantouf R.S., Budoff M.J., Ahmadi N., Ghaffari A., Flores F., Gopal A., Noori N., Jing J., Kovesdy C.P., Kalantar-Zadeh K. (2010). Total and individual coronary artery calcium scores as independent predictors of mortality in hemodialysis patients. Am. J. Nephrol..

[7] Day A.J., Prestwich G.D. (2002). Hyaluronan-binding proteins: Tying up the giant. J. Biol. Chem..

[8] Fraser J.R., Laurent T.C., Laurent U.B. (1997). Hyaluronan: Its nature, distribution, functions and turnover. J. Intern. Med..

[9] Petrey A.C., de la Motte C.A. (2014). Hyaluronan, a crucial regulator of inflammation. Front. Immunol..

[10] Camenisch T.D., Spicer A.P., Brehm-Gibson T., Biesterfeldt J., Augustine M.L., Calabro A., Kubalak S., Klewer S.E., McDonald J.A. (2000). Disruption of hyaluronan synthase-2 abrogates normal cardiac morphogenesis and hyaluronan-mediated transformation of epithelium to mesenchyme. J. Clin. Investig..

[11] Cirillo N. (2023). The Hyaluronan/CD44 Axis: A Double-Edged Sword in Cancer. Int. J. Mol. Sci..

[12] Kim J., Seki E. (2023). Hyaluronan in liver fibrosis: Basic mechanisms, clinical implications, and therapeutic targets. Hepatol. Commun..

[13] Meran S., Thomas D.W., Stephens P., Enoch S., Martin J., Steadman R., Phillips A.O. (2008). Hyaluronan facilitates transforming growth factor-beta1-mediated fibroblast proliferation. J. Biol. Chem..

[14] Midgley A.C., Rogers M., Hallett M.B., Clayton A., Bowen T., Phillips A.O., Steadman R. (2013). Transforming growth factor-beta1 (TGF-beta1)-stimulated fibroblast to myofibroblast differentiation is mediated by hyaluronan (HA)-facilitated epidermal growth factor receptor (EGFR) and CD44 co-localization in lipid rafts. J. Biol. Chem..

[15] Simpson R.M., Meran S., Thomas D., Stephens P., Bowen T., Steadman R., Phillips A. (2009). Age-related changes in pericellular hyaluronan organization leads to impaired dermal fibroblast to myofibroblast differentiation. Am. J. Pathol..

[16] Wight T.N. (2008). Arterial remodeling in vascular disease: A key role for hyaluronan and versican. Front. Biosci..

[17] Homann S., Grandoch M., Kiene L.S., Podsvyadek Y., Feldmann K., Rabausch B., Nagy N., Lehr S., Kretschmer I., Oberhuber A. (2018). Hyaluronan synthase 3 promotes plaque inflammation and atheroprogression. Matrix Biol..

[18] Fischer J.W. (2019). Role of hyaluronan in atherosclerosis: Current knowledge and open questions. Matrix Biol..

[19] Zhu L., Li Q., Qi D., Niu F., Li Q., Yang H., Gao C. (2020). Atherosclerosis-associated endothelial cell apoptosis by miRNA let7-b-mediated downregulation of HAS-2. J. Cell. Biochem..

[20] Grandoch M., Bollyky P.L., Fischer J.W. (2018). Hyaluronan: A Master Switch Between Vascular Homeostasis and Inflammation. Circ. Res..

[21] Hartmann F., Gorski D.J., Newman A.A.C., Homann S., Petz A., Owsiany K.M., Serbulea V., Zhou Y.Q., Deaton R.A., Bendeck M. (2021). SMC-Derived Hyaluronan Modulates Vascular SMC Phenotype in Murine Atherosclerosis. Circ. Res..

[22] Viola M., Karousou E., D’Angelo M.L., Caon I., De Luca G., Passi A., Vigetti D. (2015). Regulated Hyaluronan Synthesis by Vascular Cells. Int. J. Cell Biol..

[23] Davies M.R., Hruska K.A. (2001). Pathophysiological mechanisms of vascular calcification in end-stage renal disease. Kidney Int..

[24] Wada T., McKee M.D., Steitz S., Giachelli C.M. (1999). Calcification of vascular smooth muscle cell cultures: Inhibition by osteopontin. Circ. Res..

[25] Bostrom K., Watson K.E., Horn S., Wortham C., Herman I.M., Demer L.L. (1993). Bone morphogenetic protein expression in human atherosclerotic lesions. J. Clin. Investig..

[26] Bommaya G., Meran S., Krupa A., Phillips A.O., Steadman R. (2011). Tumour necrosis factor-stimulated gene (TSG)-6 controls epithelial-mesenchymal transition of proximal tubular epithelial cells. Int. J. Biochem. Cell Biol..

[27] Martin J., Midgley A., Meran S., Woods E., Bowen T., Phillips A.O., Steadman R. (2016). Tumor Necrosis Factor-stimulated Gene 6 (TSG-6)-mediated Interactions with the Inter-alpha-inhibitor Heavy Chain 5 Facilitate Tumor Growth Factor beta1 (TGFbeta1)-dependent Fibroblast to Myofibroblast Differentiation. J. Biol. Chem..

[28] Midgley A.C., Duggal L., Jenkins R., Hascall V., Steadman R., Phillips A.O., Meran S. (2015). Hyaluronan regulates bone morphogenetic protein-7-dependent prevention and reversal of myofibroblast phenotype. J. Biol. Chem..

[29] Midgley A.C., Oltean S., Hascall V., Woods E.L., Steadman R., Phillips A.O., Meran S. (2017). Nuclear hyaluronidase 2 drives alternative splicing of CD44 pre-mRNA to determine profibrotic or antifibrotic cell phenotype. Sci. Signal..

[30] Roudaut M., Caillaud A., Souguir Z., Bray L., Girardeau A., Rimbert A., Croyal M., Lambert G., Patitucci M., Delpouve G. (2024). Human induced pluripotent stem cells-derived liver organoids grown on a Biomimesys(R) hyaluronic acid-based hydroscaffold as a new model for studying human lipoprotein metabolism. Bioeng. Transl. Med..

[31] Rosales P., Vitale D., Icardi A., Sevic I., Alaniz L. (2024). Role of Hyaluronic acid and its chemical derivatives in immunity during homeostasis, cancer and tissue regeneration. Semin. Immunopathol..

[32] Ghaleno L.R., Hajari M.A., Choshali M.A., Heidari E.A., Shahverdi A., Alipour H., Valojerdi M.R. (2024). Hyaluronic acid-alginate hydrogel stimulates the differentiation of neonatal mouse testicular cells into hepatocyte-like and other cell lineages in three-dimensional culture. Biol. Cell.

[33] Simpson M.A., Heldin P. (2014). Hyaluronan signaling and turnover. Preface. Adv. Cancer Res..

[34] Kim H.R., Wheeler M.A., Wilson C.M., Iida J., Eng D., Simpson M.A., McCarthy J.B., Bullard K.M. (2004). Hyaluronan facilitates invasion of colon carcinoma cells in vitro via interaction with CD44. Cancer Res..

[35] McAtee C.O., Barycki J.J., Simpson M.A. (2014). Emerging roles for hyaluronidase in cancer metastasis and therapy. Adv. Cancer Res..

[36] Kaesler N., Schurgers L.J., Floege J. (2021). Vitamin K and cardiovascular complications in chronic kidney disease patients. Kidney Int..

[37] Levy D.S., Grewal R., Le T.H. (2020). Vitamin K deficiency: An emerging player in the pathogenesis of vascular calcification and an iatrogenic consequence of therapies in advanced renal disease. Am. J. Physiol. Ren. Physiol..

[38] Alappan H.R., Kaur G., Manzoor S., Navarrete J., O’Neill W.C. (2020). Warfarin Accelerates Medial Arterial Calcification in Humans. Arter. Thromb. Vasc. Biol..

[39] Kruger T., Oelenberg S., Kaesler N., Schurgers L.J., van de Sandt A.M., Boor P., Schlieper G., Brandenburg V.M., Fekete B.C., Veulemans V. (2013). Warfarin induces cardiovascular damage in mice. Arter. Thromb. Vasc. Biol..

[40] Neradova A., Wasilewski G., Prisco S., Leenders P., Caron M., Welting T., van Rietbergen B., Kramann R., Floege J., Vervloet M.G. (2022). Combining phosphate binder therapy with vitamin K2 inhibits vascular calcification in an experimental animal model of kidney failure. Nephrol. Dial. Transpl..

[41] Midgley A.C., Woods E.L., Jenkins R.H., Brown C., Khalid U., Chavez R., Hascall V., Steadman R., Phillips A.O., Meran S. (2020). Hyaluronidase-2 Regulates RhoA Signaling, Myofibroblast Contractility, and Other Key Profibrotic Myofibroblast Functions. Am. J. Pathol..

[42] Levin N.W., Hoenich N.A. (2001). Consequences of hyperphosphatemia and elevated levels of the calcium-phosphorus product in dialysis patients. Curr. Opin. Nephrol. Hypertens..

[43] Baune B.T., Rothermundt M., Ladwig K.H., Meisinger C., Berger K. (2011). Systemic inflammation (Interleukin 6) predicts all-cause mortality in men: Results from a 9-year follow-up of the MEMO Study. Age.

[44] Lopez-Mejias R., Gonzalez-Gay M.A. (2019). IL-6: Linking chronic inflammation and vascular calcification. Nat. Rev. Rheumatol..

[45] Kultti A., Pasonen-Seppanen S., Jauhiainen M., Rilla K.J., Karna R., Pyoria E., Tammi R.H., Tammi M.I. (2009). 4-Methylumbelliferone inhibits hyaluronan synthesis by depletion of cellular UDP-glucuronic acid and downregulation of hyaluronan synthase 2 and 3. Exp. Cell Res..

[46] Kuipers H.F., Nagy N., Ruppert S.M., Sunkari V.G., Marshall P.L., Gebe J.A., Ishak H.D., Keswani S.G., Bollyky J., Frymoyer A.R. (2016). The pharmacokinetics and dosing of oral 4-methylumbelliferone for inhibition of hyaluronan synthesis in mice. Clin. Exp. Immunol..

[47] Mello L.V., De Groot B.L., Li S., Jedrzejas M.J. (2002). Structure and flexibility of Streptococcus agalactiae hyaluronate lyase complex with its substrate. Insights into the mechanism of processive degradation of hyaluronan. J. Biol. Chem..

[48] Itano N., Kimata K. (2002). Mammalian hyaluronan synthases. IUBMB Life.

[49] Rensen S.S., Doevendans P.A., van Eys G.J. (2007). Regulation and characteristics of vascular smooth muscle cell phenotypic diversity. Neth. Heart J..

[50] Chow G., Tauler J., Mulshine J.L. (2010). Cytokines and growth factors stimulate hyaluronan production: Role of hyaluronan in epithelial to mesenchymal-like transition in non-small cell lung cancer. J. Biomed. Biotechnol..

[51] de la Motte C.A., Hascall V.C., Drazba J., Bandyopadhyay S.K., Strong S.A. (2003). Mononuclear leukocytes bind to specific hyaluronan structures on colon mucosal smooth muscle cells treated with polyinosinic acid:polycytidylic acid: Inter-alpha-trypsin inhibitor is crucial to structure and function. Am. J. Pathol..

[52] Itano N., Sawai T., Yoshida M., Lenas P., Yamada Y., Imagawa M., Shinomura T., Hamaguchi M., Yoshida Y., Ohnuki Y. (1999). Three isoforms of mammalian hyaluronan synthases have distinct enzymatic properties. J. Biol. Chem..

[53] Spicer A.P., McDonald J.A. (1998). Characterization and molecular evolution of a vertebrate hyaluronan synthase gene family. J. Biol. Chem..

[54] Axelgaard Lorenstzen K., Chai S., Chen H., Danielsen C.C., Simonsen U., Wogensen L. (2016). Mechanisms involved in extracellular matrix remodeling and arterial stiffness induced by hyaluronan accumulation. Atherosclerosis.

[55] Selbi W., de la Motte C., Hascall V., Phillips A. (2004). BMP-7 modulates hyaluronan-mediated proximal tubular cell-monocyte interaction. J. Am. Soc. Nephrol..

[56] Zhang X.L., Selbi W., de la Motte C., Hascall V., Phillips A.O. (2005). Bone morphogenic protein-7 inhibits monocyte-stimulated TGF-beta1 generation in renal proximal tubular epithelial cells. J. Am. Soc. Nephrol..

[57] Selbi W., de la Motte C.A., Hascall V.C., Day A.J., Bowen T., Phillips A.O. (2006). Characterization of hyaluronan cable structure and function in renal proximal tubular epithelial cells. Kidney Int..

[58] Meng F., Yang Z., Dianbo L., Gu M., Shang M., Zeng A., Wen X., Xue Y., Zhao X., He A. (2022). Hyaluronan size alters chondrogenesis of mesenchymal stem cells cultured on tricalcium phosphate-collagen-hyaluronan scaffolds. J. Biomed. Mat. Res..

[59] Li T., Wang Y., Tu W.P. (2023). Vitamin K supplementation and vascular calcification: A systematic review and meta-analysis of randomized controlled trials. Front. Nutr..

