# Supplementary material for: Hyaluronan Regulates Vascular Smooth Muscle Cell Osteogenic Differentiation and Vascular Calcification
Source: Biomolecules. 2026 May 15;16(5):729. doi: 10.3390/biom16050729 (PMC13204215; doi:10.3390/biom16050729)
Supplement: Supplementary file 1 [file biomolecules-16-00729-s001.zip › Meran Supplementary Data Biomolecules 2026.pptx]

## Slide 1
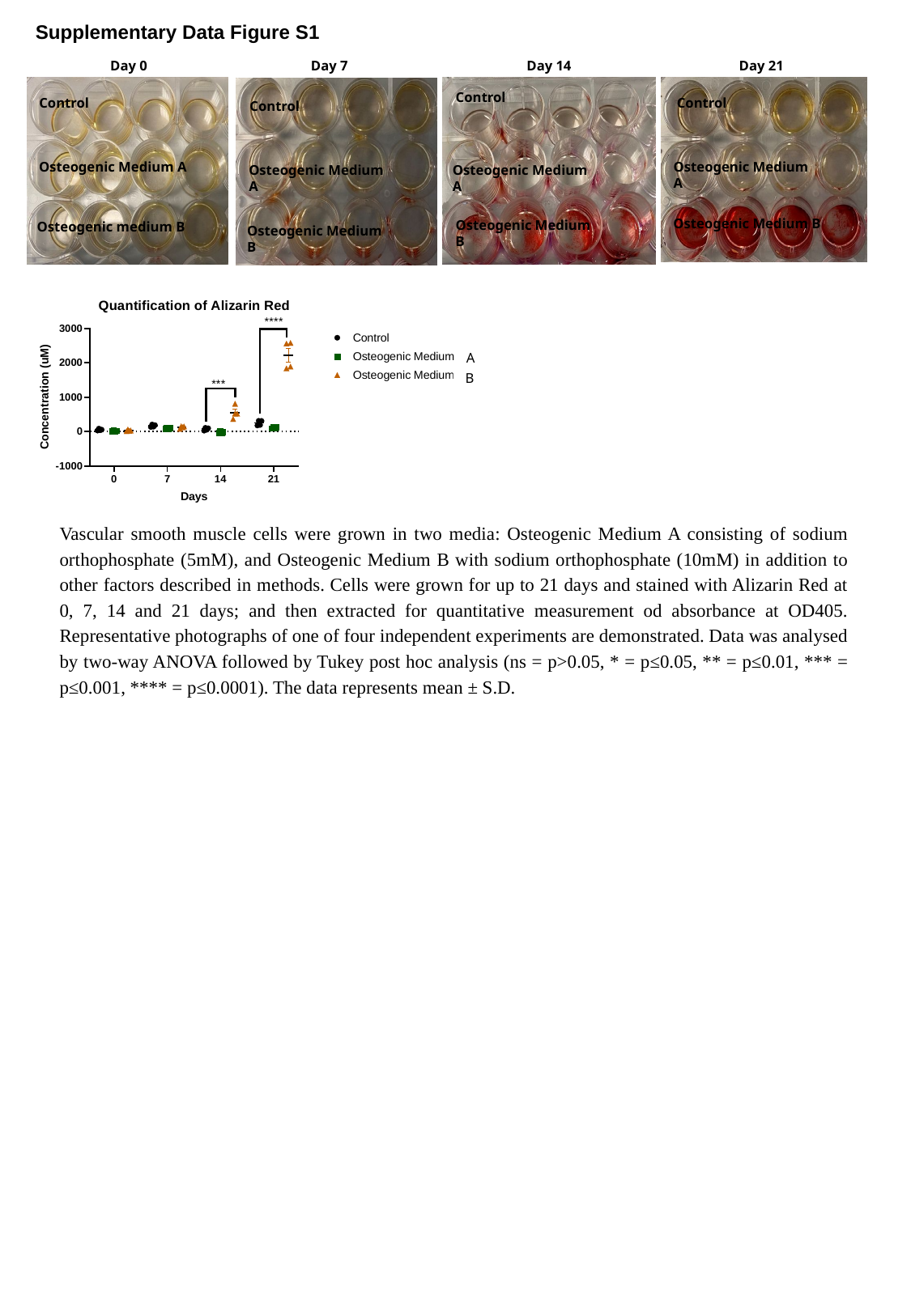

Supplementary Data Figure S1
Day 0
Day 7
Day 14
Day 21
Control
Control
Control
Control
Osteogenic Medium A
Osteogenic Medium A
Osteogenic Medium A
Osteogenic Medium A
Osteogenic Medium B
Osteogenic Medium B
Osteogenic medium B
Osteogenic Medium B
A
B
Vascular smooth muscle cells were grown in two media: Osteogenic Medium A consisting of sodium orthophosphate (5mM), and Osteogenic Medium B with sodium orthophosphate (10mM) in addition to other factors described in methods. Cells were grown for up to 21 days and stained with Alizarin Red at 0, 7, 14 and 21 days; and then extracted for quantitative measurement od absorbance at OD405. Representative photographs of one of four independent experiments are demonstrated. Data was analysed by two-way ANOVA followed by Tukey post hoc analysis (ns = p>0.05, * = p≤0.05, ** = p≤0.01, *** = p≤0.001, **** = p≤0.0001). The data represents mean ± S.D.

## Slide 2
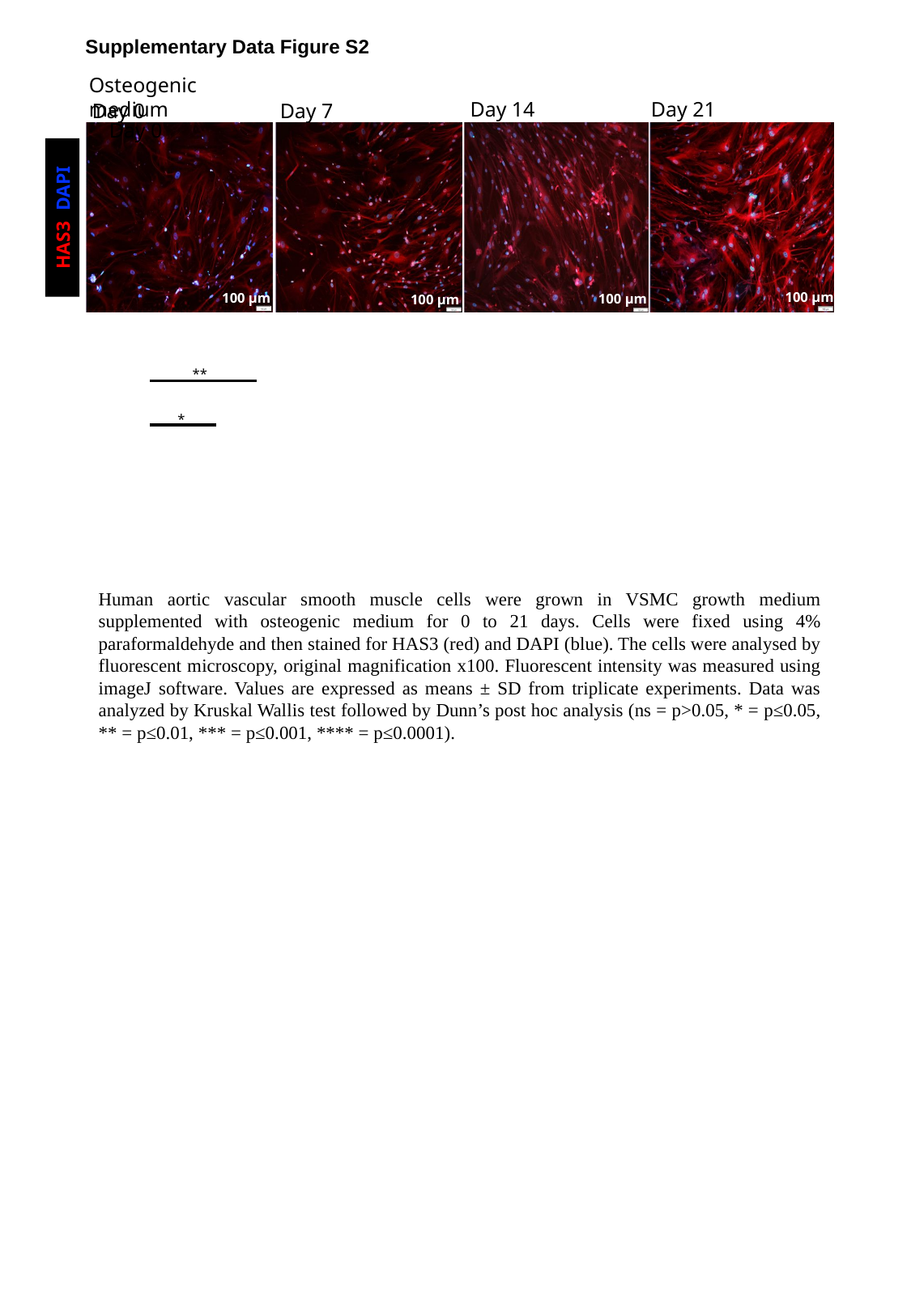

Supplementary Data Figure S2
Osteogenic medium
Day 21
Day 14
Day 0
Day 7
Day 0
HAS3, DAPI
100 μm
100 μm
100 μm
100 μm
**
*
Human aortic vascular smooth muscle cells were grown in VSMC growth medium supplemented with osteogenic medium for 0 to 21 days. Cells were fixed using 4% paraformaldehyde and then stained for HAS3 (red) and DAPI (blue). The cells were analysed by fluorescent microscopy, original magnification x100. Fluorescent intensity was measured using imageJ software. Values are expressed as means ± SD from triplicate experiments. Data was analyzed by Kruskal Wallis test followed by Dunn’s post hoc analysis (ns = p>0.05, * = p≤0.05, ** = p≤0.01, *** = p≤0.001, **** = p≤0.0001).

## Slide 3
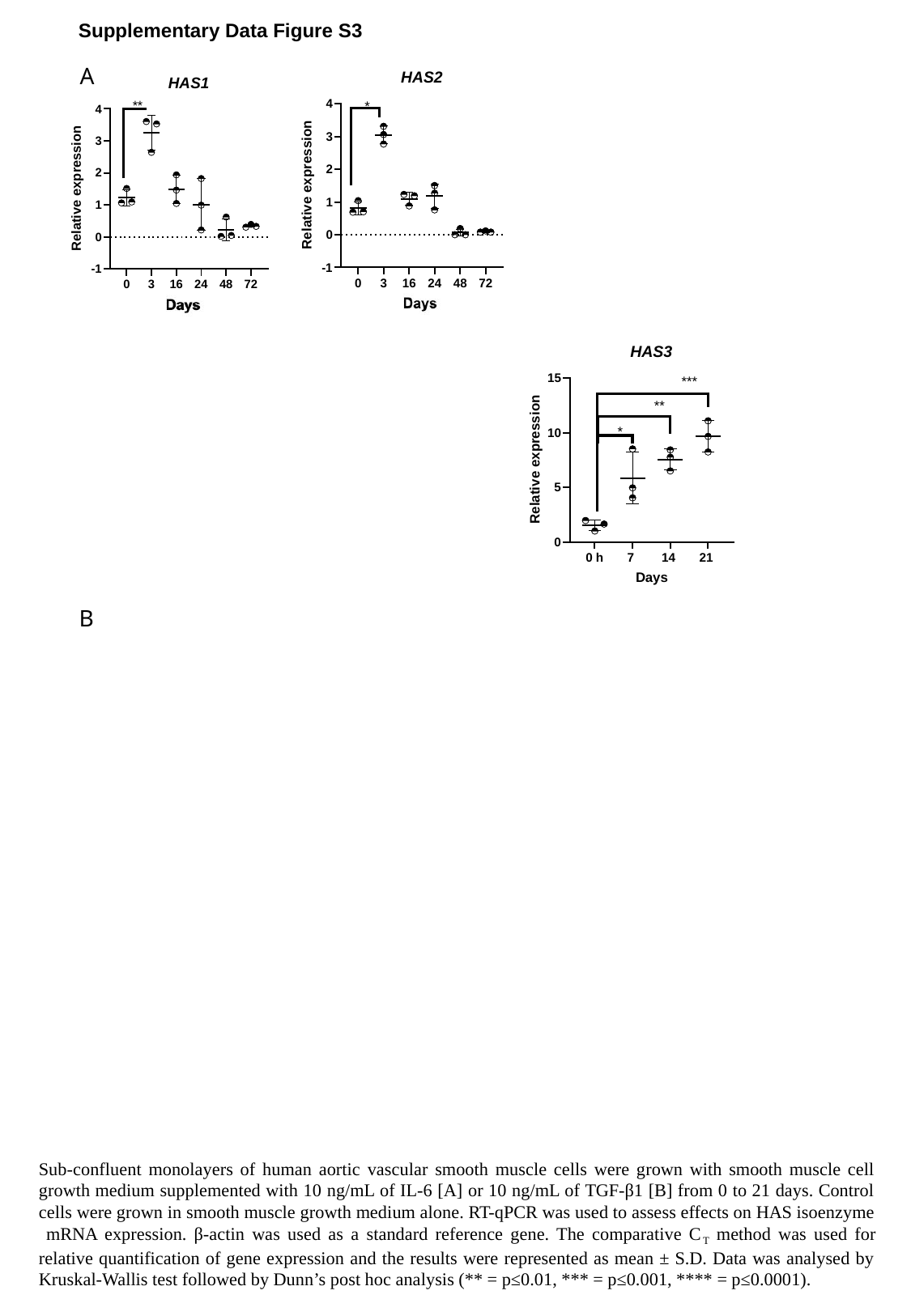

Supplementary Data Figure S3
A
B
Sub-confluent monolayers of human aortic vascular smooth muscle cells were grown with smooth muscle cell growth medium supplemented with 10 ng/mL of IL-6 [A] or 10 ng/mL of TGF-β1 [B] from 0 to 21 days. Control cells were grown in smooth muscle growth medium alone. RT-qPCR was used to assess effects on HAS isoenzyme mRNA expression. β-actin was used as a standard reference gene. The comparative CT method was used for relative quantification of gene expression and the results were represented as mean ± S.D. Data was analysed by Kruskal-Wallis test followed by Dunn’s post hoc analysis (** = p≤0.01, *** = p≤0.001, **** = p≤0.0001).

## Slide 4
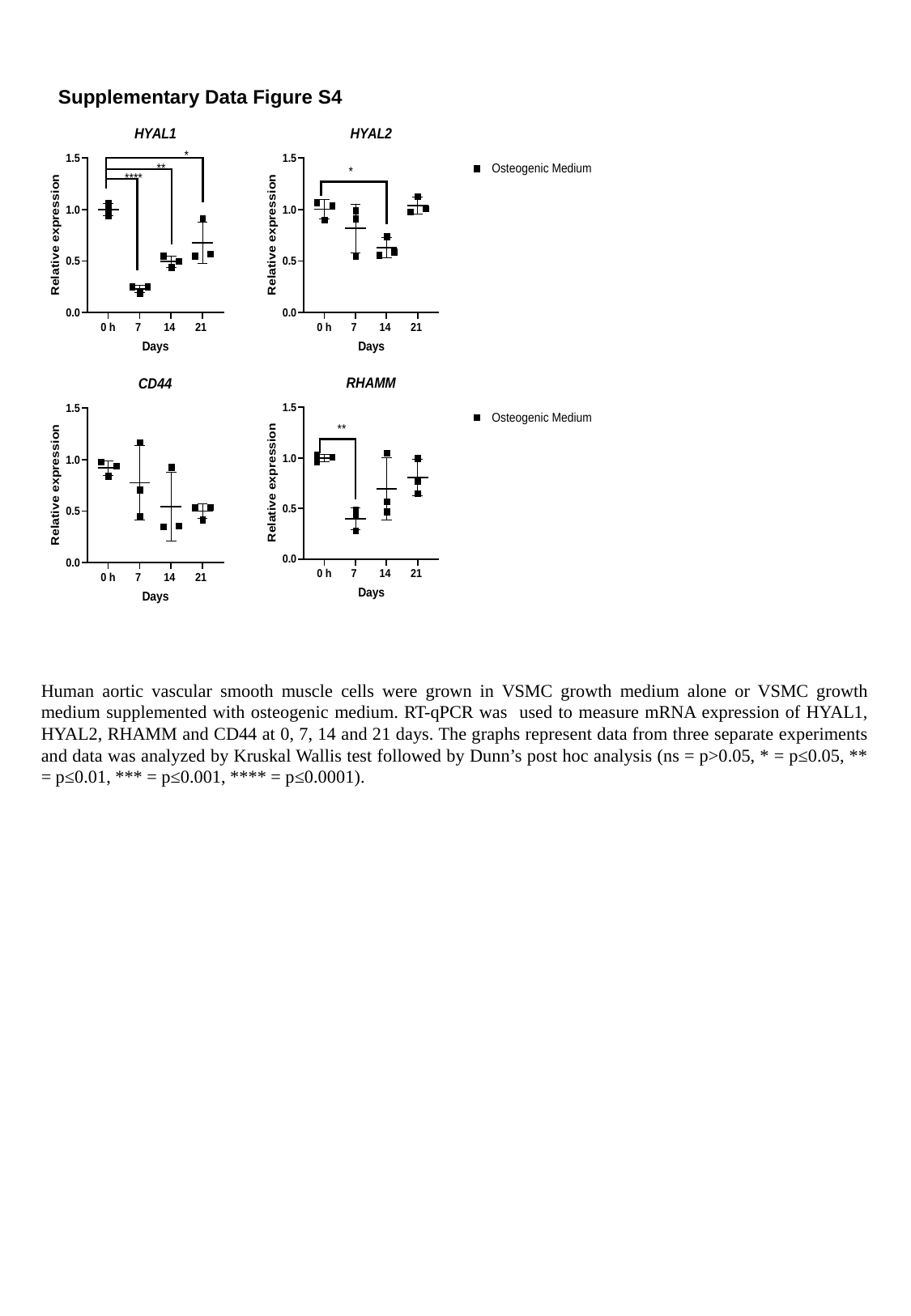

Supplementary Data Figure S4
Human aortic vascular smooth muscle cells were grown in VSMC growth medium alone or VSMC growth medium supplemented with osteogenic medium. RT-qPCR was used to measure mRNA expression of HYAL1, HYAL2, RHAMM and CD44 at 0, 7, 14 and 21 days. The graphs represent data from three separate experiments and data was analyzed by Kruskal Wallis test followed by Dunn’s post hoc analysis (ns = p>0.05, * = p≤0.05, ** = p≤0.01, *** = p≤0.001, **** = p≤0.0001).

## Slide 5
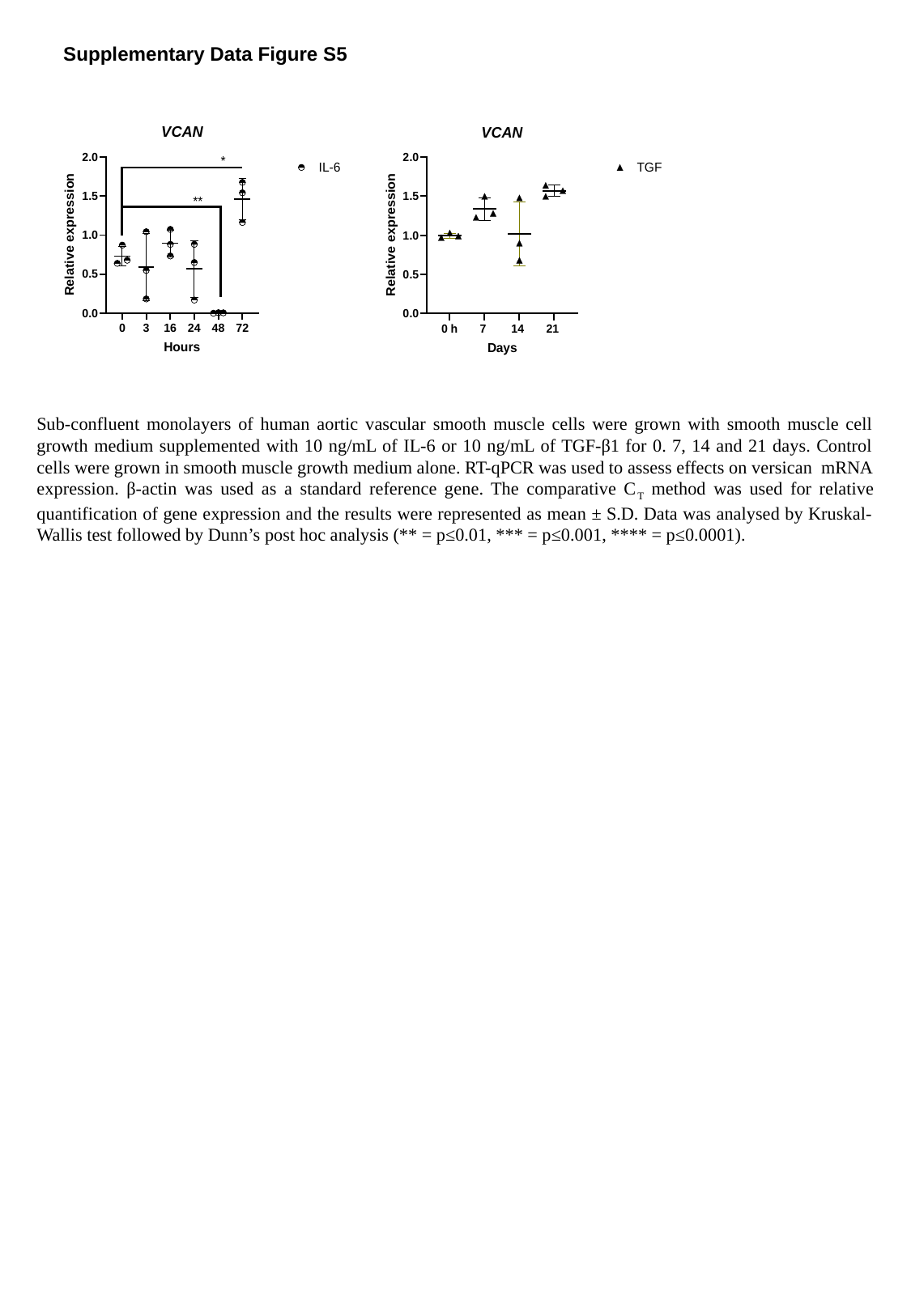

Supplementary Data Figure S5
Sub-confluent monolayers of human aortic vascular smooth muscle cells were grown with smooth muscle cell growth medium supplemented with 10 ng/mL of IL-6 or 10 ng/mL of TGF-β1 for 0. 7, 14 and 21 days. Control cells were grown in smooth muscle growth medium alone. RT-qPCR was used to assess effects on versican mRNA expression. β-actin was used as a standard reference gene. The comparative CT method was used for relative quantification of gene expression and the results were represented as mean ± S.D. Data was analysed by Kruskal-Wallis test followed by Dunn’s post hoc analysis (** = p≤0.01, *** = p≤0.001, **** = p≤0.0001).

## Slide 6
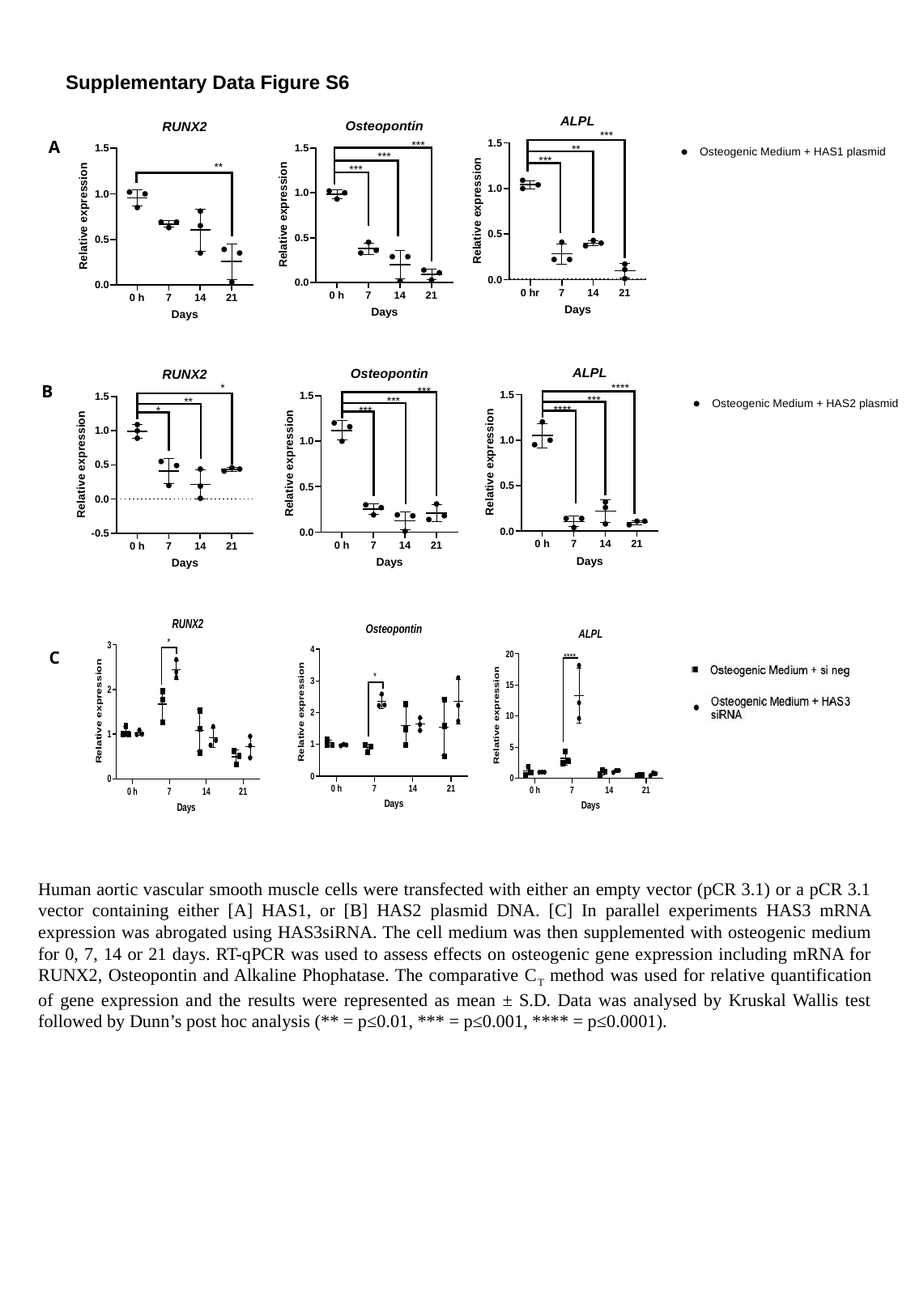

Supplementary Data Figure S6
A
B
C
Human aortic vascular smooth muscle cells were transfected with either an empty vector (pCR 3.1) or a pCR 3.1 vector containing either [A] HAS1, or [B] HAS2 plasmid DNA. [C] In parallel experiments HAS3 mRNA expression was abrogated using HAS3siRNA. The cell medium was then supplemented with osteogenic medium for 0, 7, 14 or 21 days. RT-qPCR was used to assess effects on osteogenic gene expression including mRNA for RUNX2, Osteopontin and Alkaline Phophatase. The comparative CT method was used for relative quantification of gene expression and the results were represented as mean ± S.D. Data was analysed by Kruskal Wallis test followed by Dunn’s post hoc analysis (** = p≤0.01, *** = p≤0.001, **** = p≤0.0001).

## Slide 7
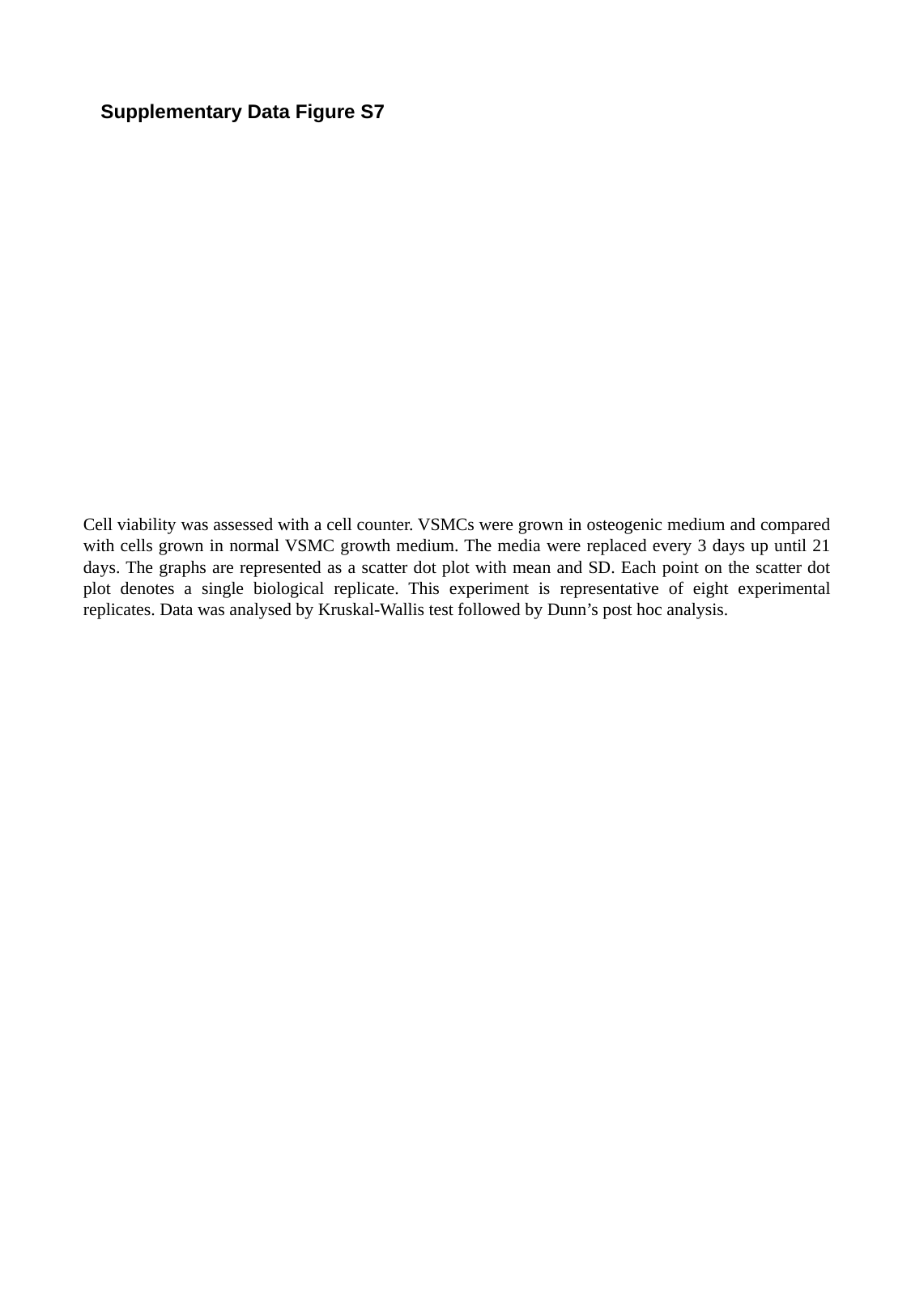

Supplementary Data Figure S7
Cell viability was assessed with a cell counter. VSMCs were grown in osteogenic medium and compared with cells grown in normal VSMC growth medium. The media were replaced every 3 days up until 21 days. The graphs are represented as a scatter dot plot with mean and SD. Each point on the scatter dot plot denotes a single biological replicate. This experiment is representative of eight experimental replicates. Data was analysed by Kruskal-Wallis test followed by Dunn’s post hoc analysis.

## Slide 8
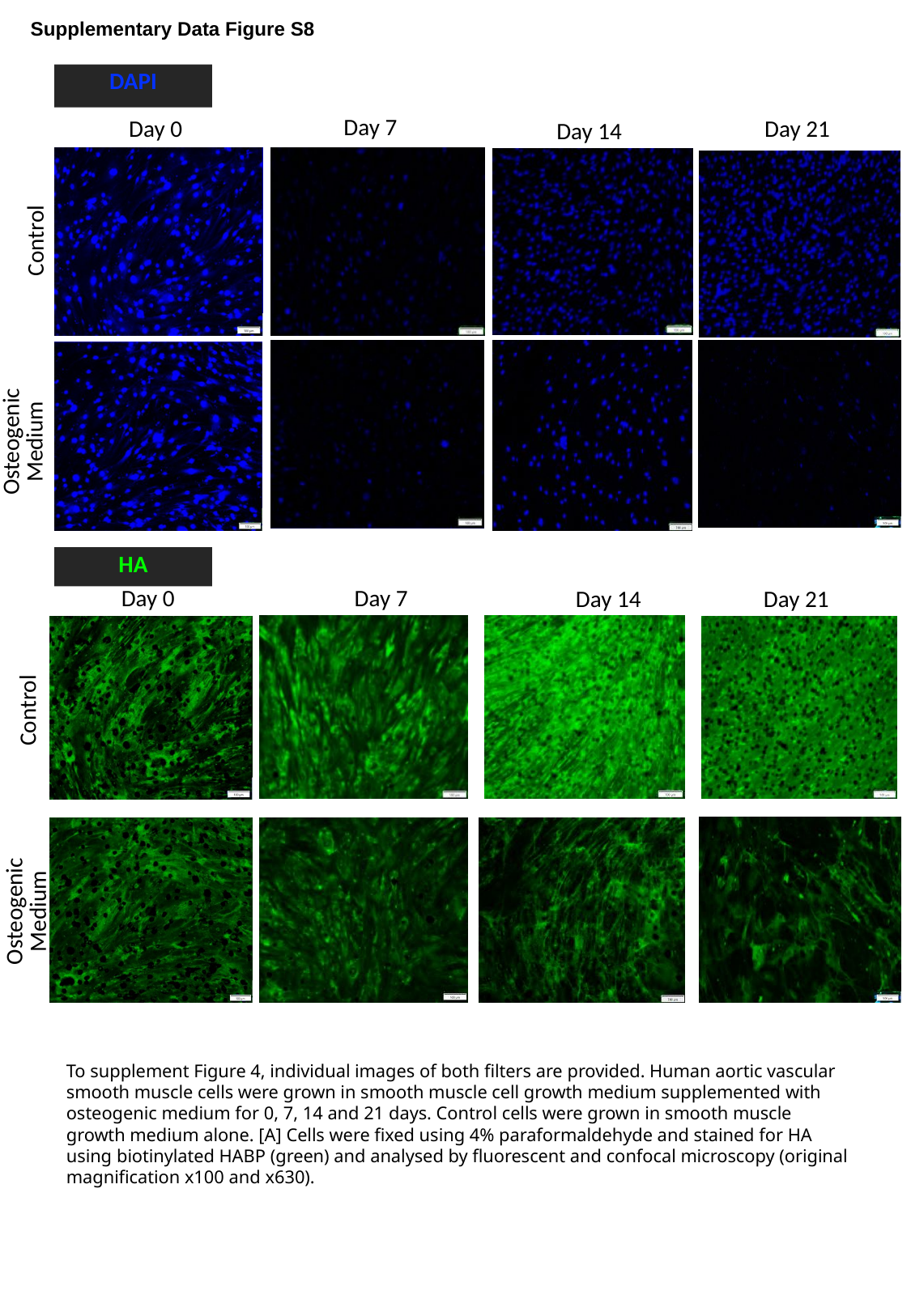

Supplementary Data Figure S8
DAPI
Day 7
Day 0
Day 21
Day 14
Control
Osteogenic Medium
HA
Day 0
Day 7
Day 14
Day 21
Control
Osteogenic Medium
To supplement Figure 4, individual images of both filters are provided. Human aortic vascular smooth muscle cells were grown in smooth muscle cell growth medium supplemented with osteogenic medium for 0, 7, 14 and 21 days. Control cells were grown in smooth muscle growth medium alone. [A] Cells were fixed using 4% paraformaldehyde and stained for HA using biotinylated HABP (green) and analysed by fluorescent and confocal microscopy (original magnification x100 and x630).
